# Social network data from teacher leader development

**DOI:** 10.1016/j.dib.2019.104182

**Published:** 2019-06-24

**Authors:** Samuel J. Polizzi, Brandon Ofem, William Coyle, Keith Lundquist, Gregory T. Rushton

**Affiliations:** a Department of Chemistry and Tennessee STEM Education Center, Middle Tennessee State University, Murfreesboro, TN 37130, USA; b Department of Chemistry and Biochemistry, Kennesaw State University, Kennesaw, GA 30144, USA; c Department of Global Leadership and Management, University of Missouri – St. Louis, St. Louis, MO 63121, USA; d Department of Chemistry and Institute for STEM Education, Stony Brook University, Stony Brook, NY 11790, USA

**Keywords:** Social network analysis, Visual network scales, Teacher leadership, Science teacher education, Professional development

## Abstract

Social network analysis can draw upon surveys and discussions to generate quantitative and qualitative data. We describe network data generated via a social network survey and discussion activity with high school science teachers in a teacher leadership development program. Data include social network maps related to seeking expertise in teaching content and/or pedagogy, disaggregated by contacts at the school, district, state, nation, and international spheres of influence. Data also include transcripts of the activity and teacher discussions of networks in their own educational settings. This data article is related to the research article, “The use of visual network scales in teacher leader development” Polizzi et al., 2019, where data interpretation can be found.

**Table:** Specifications table

Subject area	*Social Network Analysis, Education*
More specific subject area	*Visual Network Scales, Science Teacher Leadership*
Type of data	*Table, figure, survey instrument, activity slides, transcripts*
How data was acquired	*Researcher made survey instrument, Transcript of activity video*
Data format	*Raw, analyzed, descriptive data*
Experimental factors	*Self-reported contacts and interactions converted to network maps in UCINET and NETDRAW, transcript of activity anonymized*
Experimental features	*Network maps analyzed for size, density, spheres of influence; transcripts examined for network themes*
Data source location	*University setting in Southeastern region of United States*
Data accessibility	*Data is with this article*
Related research article	*Companion paper to research article**[1]**S.J. Polizzi, B. Ofem, W. Coyle, K. Lundquist, G.T. Rushton, The use of visual network scales in teacher leader development, Teaching and Teacher Education, 83 (2019) 42–53.*

**Table:** 

**Value of the data** • Social network analysis is crossing fields out of organizational behavior, management, and social science, and of growing interest to the fields of science and education. • Examples of the instruments and logic used to generate social network data are not widely available and should be shared to promote access to social network analysis. • Social network data and network discussions are rich and can be analyzed through different lenses, such as identity or communities of practice, when data are made available. • Education studies are often highly contextual and local to a single site, but data availability can facilitate meta-analyses for more generalizable findings across science disciplines or geography.

## 1 Data

**Fig. 1:**
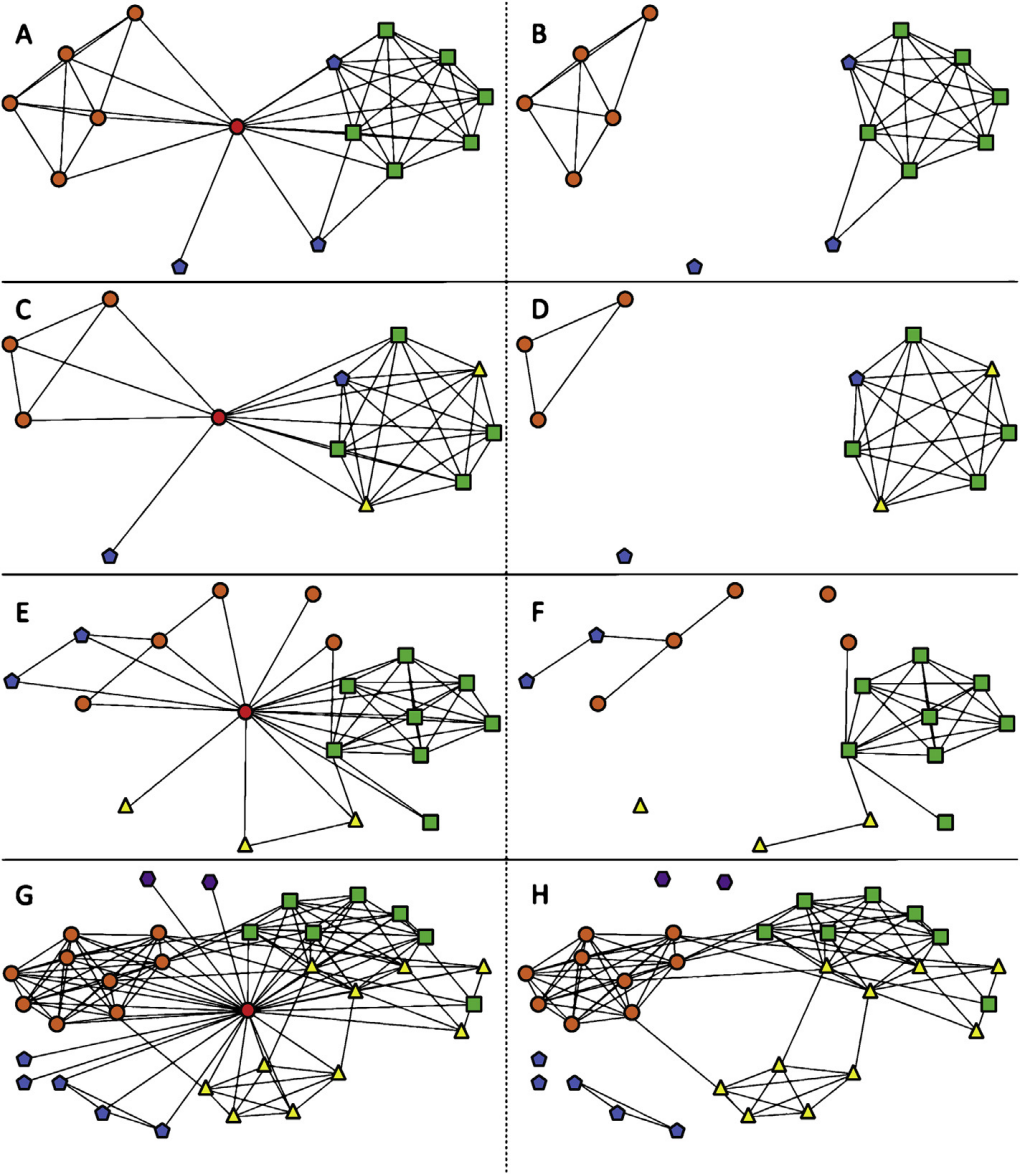
Ego network diagrams 1–4 of 13 in sample. The leftmost column depicts all reported interactions centered on the ego. The rightmost column depicts the same network, but the ego has been removed to more easily visualize the isolated contacts and more densely connected groups of contacts. Nodes are colored and shaped by closest geography relative to the ego (red circle) to show contacts in the same school (orange circle), district (yellow triangle), state (green square), nation (blue pentagon) or international (purple hexagon), noting that a teacher at the same school is listed only at the school level, despite being in the same district, state and nation.

**Fig. 2:**
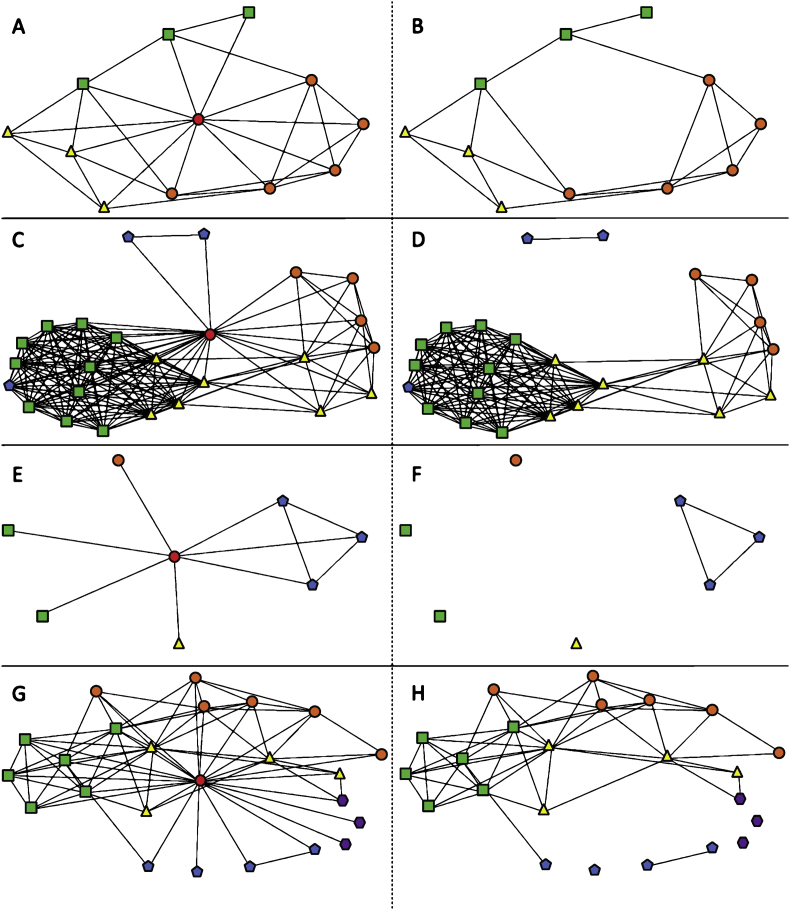
Ego network diagrams 5–8 of 13 in sample. The leftmost column depicts all reported interactions centered on the ego. The rightmost column depicts the same network, but the ego has been removed to more easily visualize the isolated contacts and more densely connected groups of contacts. Nodes are colored and shaped by closest geography relative to the ego (red circle) to show contacts in the same school (orange circle), district (yellow triangle), state (green square), nation (blue pentagon) or international (purple hexagon), noting that a teacher at the same school is listed only at the school level, despite being in the same district, state and nation.

**Fig. 3:**
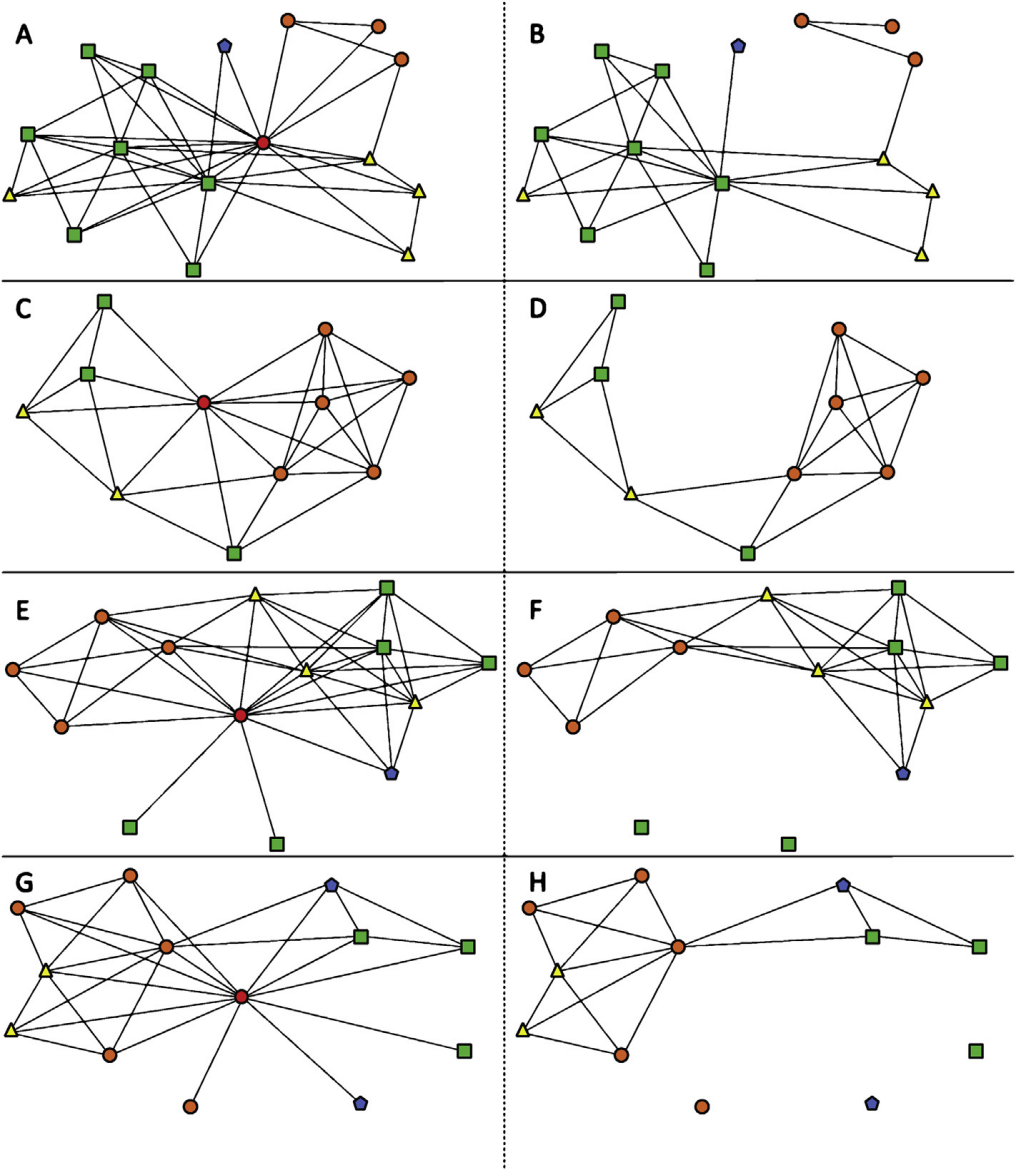
Ego network diagrams 9–12 of 13 in sample. The leftmost column depicts all reported interactions centered on the ego. The rightmost column depicts the same network, but the ego has been removed to more easily visualize the isolated contacts and more densely connected groups of contacts. Nodes are colored and shaped by closest geography relative to the ego (red circle) to show contacts in the same school (orange circle), district (yellow triangle), state (green square), nation (blue pentagon) or international (purple hexagon), noting that a teacher at the same school is listed only at the school level, despite being in the same district, state and nation.

**Fig. 4:**
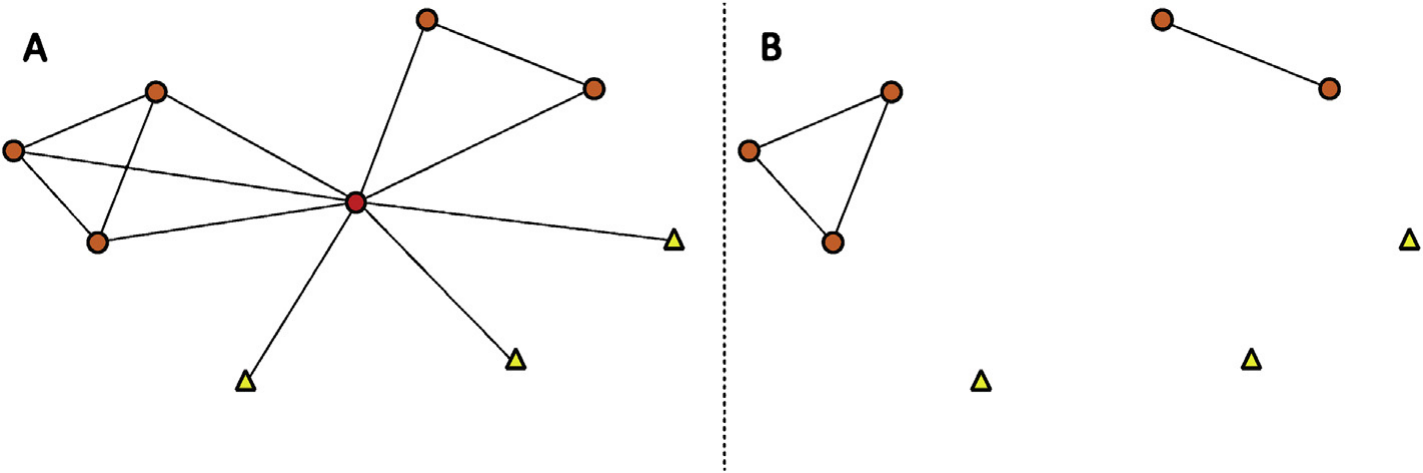
Ego network diagram 13 of 13 in sample. The leftmost column depicts all reported interactions centered on the ego. The rightmost column depicts the same network, but the ego has been removed to more easily visualize the isolated contacts and more densely connected groups of contacts. Nodes are colored and shaped by closest geography relative to the ego (red circle) to show contacts in the same school (orange circle), district (yellow triangle), state (green square), nation (blue pentagon) or international (purple hexagon), noting that a teacher at the same school is listed only at the school level, despite being in the same district, state and nation.

Data files included in this article are social network maps from an online network survey instrument, and transcript data from a guided activity instrument. Below, Fig. 1, Fig. 2, Fig. 3, Fig. 4 depict a total of 13 teacher (i.e. ego) network maps with the survey taker in the middle (left panel), and also without the survey taker to illustrate isolated contacts in the network (right panel). Network contacts are coded by color and shape for geographic spheres of influence relative to the ego. The social network survey instrument (.DOCX) used to collect teacher network contacts, along with researcher annotations/comments, is provided in Appendix A.

Seventeen (17) pages of transcript data (.DOCX) are provided in Appendix B. Names have been removed for participant anonymity, and replaced with “Facilitator”, or sequentially numbered by participant gender for female speakers “FS #” and male speakers “MS #”. The transcription service excluded “um” and pauses from the transcript unless appearing to provide emphasis. Transcripts include statements marked as unintelligible with time stamps from the video recording. If possible, unintelligible statements were corrected by the researchers reviewing the video recording before data analysis commenced. The guided activity instrument (.DOCX) from the social network activity used to collect the transcript data is provided in Appendix C.

## 2 Experimental design, materials, and methods

### 2.1 Study details

In the related research article in *Teaching and Teacher Education*, we described our interest in the use of Visual Network Scales (VNS) and Social Network Analysis (SNA) in teacher leadership Professional Development (PD) activities [1]. Therefore, we designed a network-based leadership activity for a cohort of 16 Master Teaching Fellows (MTFs) enrolled in a National Science Foundation grant to develop science teacher leaders. MTF selection and the leadership framework have previously been described in detail [2], [3]. MTF nominations were solicited by the university grant staff from partnering school districts in a greater metro area in the southeastern US. MTF candidates were teaching full time in the classroom, had greater than 5 years of teaching experience, a Master's degree or higher, and were recognized at their school level as mid-career teachers with leadership potential, but without extensive leadership training or beginning a path to school or district administration (e.g. principal or curriculum coach). MTFs were competitively accepted based on academic transcripts, essays, face-to-face interviews, and a willingness to accept the grant requirements of attending monthly face-to-face grant meetings and continuing to teach for 5 years in a high needs school district for a $10,000/year stipend. All 16 MTFs were invited to participate in the study reported here.

At the time this study was conducted, MTFs were completing the final course in a 3-part Teacher Leader Endorsement Certification program offered by a regional education service agency recognized by their state. Within the grant, teachers were developed using a framework that combined Dempsey's [4] four metaphors for teachers as leaders (i.e. teacher as a … fully functioning person, reflective practitioner, scholar, and learning partner) with Goodwin's [5] notion of professional vision (i.e. coding, highlighting, and articulating the competencies within a profession). Within the grant, MTFs regularly engaged in discussions with each other, 16 early career Teaching Fellows, and project staff related to science education, professional identity, professional vision, and leadership plans. MTFs had high school main teaching assignments in Physics (50%) or Chemistry (50%), and 9 of 16 of MTFs identified as female (56%).

Our PD activity began with an online network survey for all 16 MTFs (see Appendix A for survey instrument). In addition to serving as a source for data collection, the SNA survey instrument provided a foundation for the PD activity. In the first part of the SNA survey, teachers were encouraged to think about professional contacts who were important enough for them to “go to” for teaching information, and supply those names or initials. Further, the contacts were solicited at multiple geographic ranges to expand the contemplation of interactions beyond a single school. Completing the survey was anticipated to prepare teachers for the PD activity, and participants were informed that the surveys would be discussed at an upcoming monthly grant meeting 1–2 weeks later.

The PD activity continued during a day-long, face-to-face meeting over the course of one (1) hour (see Appendix B for activity instrument). The activity introduced teacher participants to network principles and how the survey they had taken could be used to generate simple network maps. Although geographic sphere of influence information was captured in the survey, that school/district/state/(inter)national information was not explicitly included in images used in the activity. Instead, the activity focused on visual representations of networks with different densities, or degrees of interconnectedness between network members. Network images formed a Visual Network Scale of density, covering multiple examples along the continuum of fully dense (closed) networks and less dense (open) networks. Participants discussed two scenarios related to specific open and closed density images, and then the discussion was broadened to any examples of networks that teachers encountered in their educational settings. The PD activity was video recorded by the researchers and then transcribed by a third-party service.

### 2.2 Analysis

Social network survey data for each participant included a list of the teacher's contacts (i.e. ego network size), and which of those contacts were perceived to interact with each other (i.e. ego network density). Data were formatted as a 1-mode interaction matrix in Microsoft Excel. Survey data also included the geographic proximity of contacts relative to the participant. Geographic information [i.e. same school/district/state/(inter)national] for the participant and their contacts was formatted as a 2-mode attribute matrix in Microsoft Excel. The 1-mode interaction matrix was imported into UCINET software to calculate ego network size and density reported in the main research article [1]. The 1-mode interaction matrix and 2-mode attribute matrix for geography were imported into NETDRAW software to construct network maps that could be shared with participants during the monthly meeting. Network maps were then coded by color and shape for the geographic proximity attribute for this data report (Fig. 1, Fig. 2, Fig. 3, Fig. 4).

Transcript data from the network discussion was manually coded with *a priori* codes from the activity instrument (Appendix B) related to open and network density and associated social capital outcomes [1]. Coding occurred at the level of “comment,” referring to a teacher's spoken thoughts on the topic. This corresponds to a single sentence in some cases, or entire sections or paragraphs marked for a speaker in the transcript. Comments were long enough to determine if a teacher was only talking about open or closed networks, or if there was a longer expression of ideas on both open and closed networks. Interruptions from the group affirmative or negatives, or facilitator, did not interrupt the interpretation of a comment, which might otherwise have broken one continuous comment into separate open and closed codes and inflated the number of comments. However an entire comment might only contain brief portions of dialogue that could be coded as open or closed (see Appendix C for example comments in transcript data). We identified 28 comments for coding from 9 distinct participants in the discussion. Example comments are below.

#### 2.2.1 Example long comment

“I was going to say something along those lines. If I'm in charge of creating curriculum, then I want a network that looks like that, where I can pull from lots of resources that are going to have different information. I wouldn't want a closed network where, if one person doesn't know it, no one knows it. But if I was trying to get something done as a group, I'd probably prefer the closed group, where we're all pretty closely tied to each other and work well together.”

#### 2.2.2 Example single comment despite interruption in transcript

FS 8: “What I was thinking about with these questions was that this didn't tie into anything like the larger network. Like, you know, where would I go to get new info or new ideas would probably be to go to a conference, which would tie me into a whole other set of networks. If this is only individuals, not like connected to organizations, where you could get a whole lot of—"

Facilitator: “Hm-hmm [affirmatives].”

FS 8: “Like what we were talking about with the AP stuff earlier, the AP online resources came up. That's like that's a place to get some support as well as the new ideas. So that's something else to consider as part for your overall network, or the other network you can tap into.”

#### 2.2.3 Example short comment

“If everybody else is into doing the same thing, it might be effective to work together, you know, each of you can research different parts and bring it together into a program.”

## Figures and Tables

**Figure:**
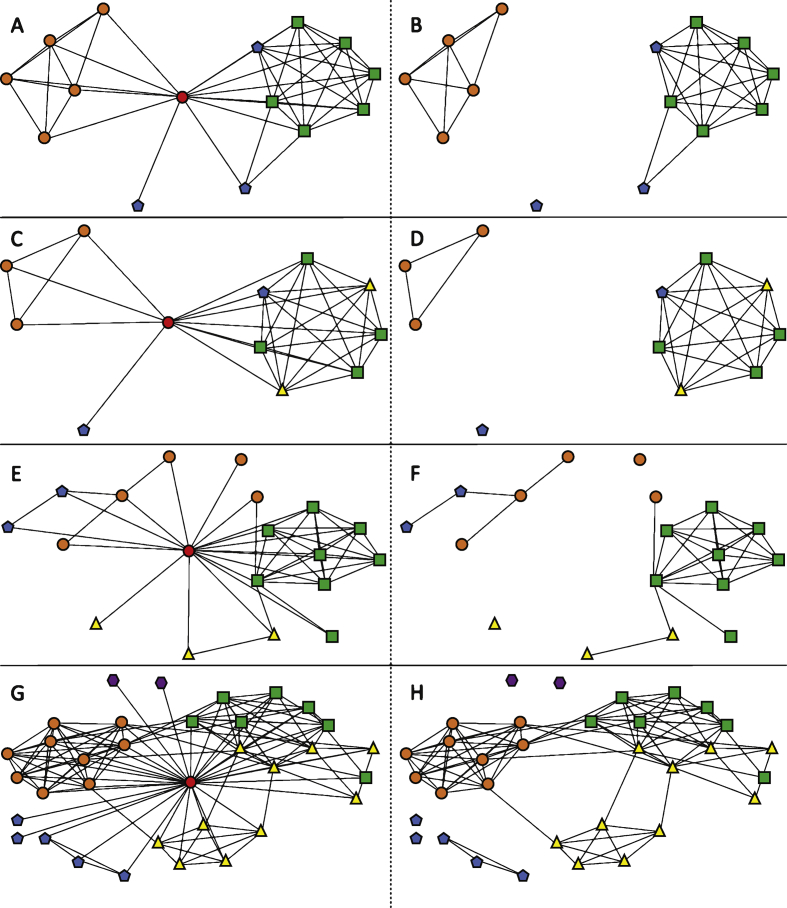


**Figure:**
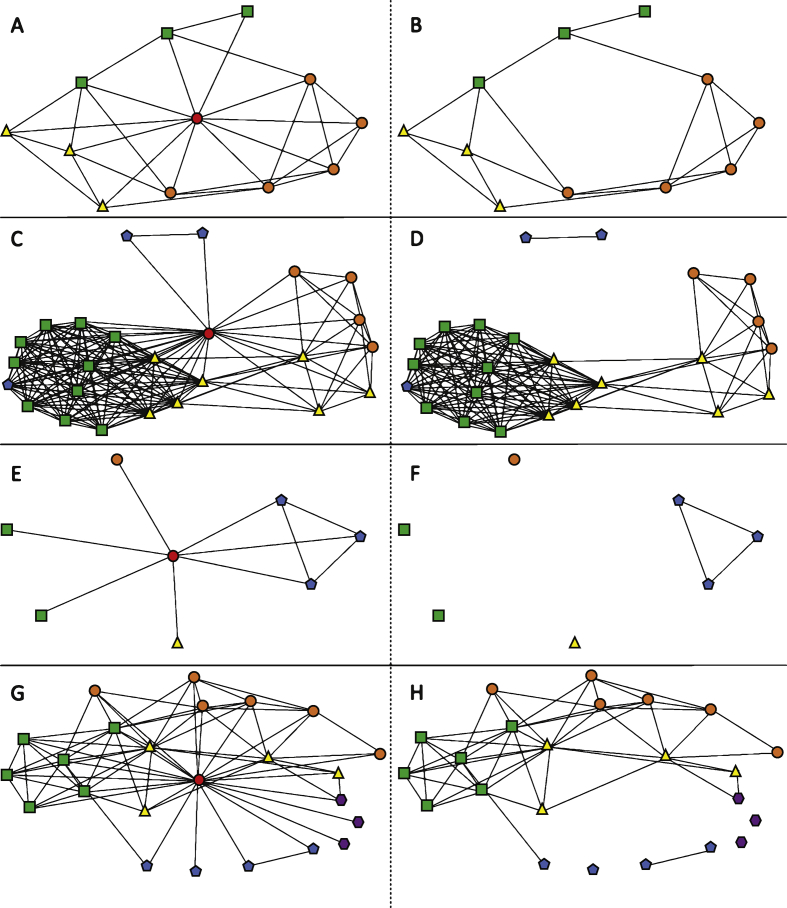


**Figure:**
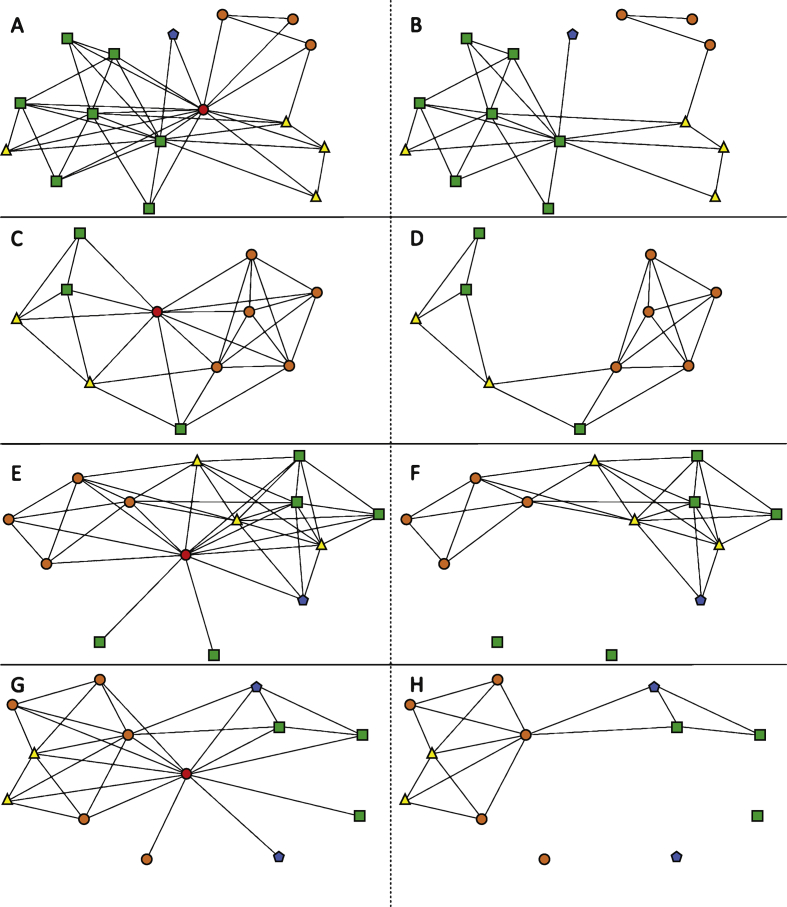


**Figure:**
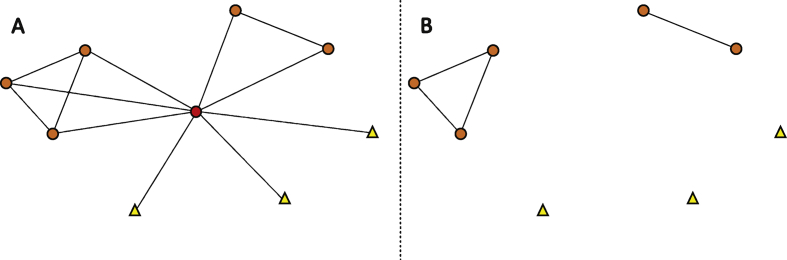


## References

[1] Polizzi S.J., Ofem B., Coyle W., Lundquist K., Rushton G.T. (2019). The use of visual network scales in teacher leader development. Teach. Teach. Educ..

[2] Criswell B.A., Rushton G.T., McDonald S.P., Gul T. (2018). A clearer vision: creating and evolving a model to support the development of science teacher leaders. Res. Sci. Educ..

[3] Criswell B.A., Rushton G.T. (2013). A Clearer Vision: Findings from the First Year of a Project Designed to Develop Teacher Leaders, Annual International Conference of the National Association for Research in Science Teaching.

[4] Dempsey R. (1992). Teachers as leaders: towards a conceptual framework, teach. Educ. Next.

[5] Goodwin C. (1994). Professional vision. Am. Anthropol..

