# Supplementary material for: Social network data from teacher leader development
Source: Data Brief. 2019 Jun 24;25:104182. doi: 10.1016/j.dib.2019.104182 (PMC6617162; doi:10.1016/j.dib.2019.104182)
Supplement: Supplementary file 1 [file mmc1.docx]

Social Network Data from Teacher Leader Development

Polizzi, SJ; Ofem, B; Coyle, W; Lundquist, K; Rushton, GT

Appendix A: Network Survey Instrument

[Notes that did not appear in the survey are provided on the following pages in red italic font for manuscript supplemental information]

Online Consent Approval

Welcome! This survey will ask some brief questions about yourself, and then ask you for the individuals you would go to for teaching-related information. Full names are encouraged but not required. If you use nicknames or abbreviations, please choose distinct identifiers that you will remember during the survey.

Contacts should be provided on different screens corresponding to their location related to you: school, district/county, state, national, and international. You will also be asked whether each individual contact interacts with your other contacts for teaching-related information.

Please note that your responses are CONFIDENTIAL. The individual results will be kept confidential, and all results will be either aggregated and/or de-identified.

Informed consent form

Consent Form

After reading the Consent Form, click on a choice below.

- ◯ I agree and give my consent to participate in this research project. I understand that participation is voluntary and that I may withdraw my consent at any time without penalty.
- ◯ I do not consent to participate in the research, but I agree to continue with the remainder of the questions for internal Noyce Program evaluation.
- ◯ I do not agree to participate in the research, and will be excluded from the remainder of the questions.

Response section

Please select whether you are a Noyce Master Teaching Fellow:

- ◯ Noyce MTF
- ◯ Not a Noyce MTF

Enter your name below: (If you do not want a followup response, you may enter a psuedonym)

[ ] ***[this is the ego in an ego network]***

Enter your years of experience below:

Teaching experience	
Formal leadership experience	

Enter your age:

Select your primary role:

- ◯ Leader or teacher of students
- ◯ Leader or teacher of teachers

Answer the question below as completely as possible. Full names are encouraged but not required. If you use nicknames or abbreviations, please choose distinct identifiers that you will remember during the survey. Contacts should be provided on different pages corresponding to their location related to you: city/school, district/county, state, national, and international. Five additional fields will appear if the initial fields are filled. Leave unused fields blank (please do not type N/A) and hit the next button to proceed to the next question.

[name generator for primary ties]

Who would you go to for information on teaching content and/or pedagogy in the school in which you work?

For example, colleagues in your department or other departments in your school. If you do not work at a school, list contacts within the office that you work.

Contact 1	***[School contact 1, and so on]***
Contact 2	
Contact 3	
Contact 4	
Contact 5	

Contact 6	
Contact 7	
Contact 8	
Contact 9	
Contact 10	

Answer the question below as completely as possible. Full names are encouraged but not required. If you use nicknames or abbreviations, please choose distinct identifiers that you will remember during the survey. Contacts should be provided on different pages corresponding to their location related to you: city/school, district/county, state, national, and international. Five additional fields will appear if the initial fields are filled. Leave unused fields blank (please do not type N/A) and hit the next button to proceed to the next question.

Who would you go to for information on teaching content and/or pedagogy in the district/county in which you work?
For example, colleagues at other nearby schools or the district office.

Contact 1	*[District contact 1 and so on]*
Contact 2	
Contact 3	
Contact 4	
Contact 5	

Contact 6

Contact 7

Contact 8

Contact 9

Contact 10

Answer the question below as completely as possible. Full names are encouraged but not required. If you use nicknames or abbreviations, please choose distinct identifiers that you will remember during the survey. Contacts should be provided on different pages corresponding to their location related to you: city/school, district/county, state, national, and international. Five additional fields will appear if the initial fields are filled. Leave unused fields blank (please do not type N/A) and hit the next button to proceed to the next question.

Who would you go to for information on teaching content and/or pedagogy in the state in which you work?

For example, colleagues at the state board of education or high schools and colleges outside your county.

Contact 1 *[State contact 1 and so on]*

Contact 2

Contact 3

Contact 4

Contact 5

Contact 6

Contact 7

Contact 8

Contact 9

Contact 10

Answer the question below as completely as possible. Full names are encouraged but not required. If you use nicknames or abbreviations, please choose distinct identifiers that you will remember during the survey. Contacts should be provided on different pages corresponding to their location related to you: city/school, district/county, state, national, and international. Five additional fields will appear if the initial fields are filled. Leave unused fields blank (please do not type N/A) and hit the next button to proceed to the next question.

Who would you go to for information on teaching content and/or pedagogy in a different state within the nation in which you work?
For example, colleagues working primarily in other states regardless of position.

Contact 1 ***[National contact 1 and so on]***

Contact 2

Contact 3

Contact 4

Contact 5

Contact 6

Contact 7

Contact 8

Contact 9

Contact 10

Answer the question below as completely as possible. Full names are encouraged but not required. If you use nicknames or abbreviations, please choose distinct identifiers that you will remember during the survey. Contacts should be provided on different pages corresponding to their location related to you: city/school, district/county, state, national, and international. Five additional fields will appear if the initial fields are filled. Leave unused fields blank (please do not type N/A) and hit the next button to proceed to the next question.

Who would you go to for information on teaching content and/or pedagogy outside the country in which you work?

For example, international colleagues or online-based contacts residing overseas.

Contact 1 ***[International contact 1 and so on]***

Contact 2

Contact 3

Contact 4

Contact 5

Contact 6	
Contact 7	
Contact 8	
Contact 9	
Contact 10	

Network Ties

[The internal mechanics of the survey inserts the names provided on previous pages into this selection list]

Select the contact(s) from the list below that apply to the following question. Do not select the contact for themselves. Click the next button to proceed to the next question. *[alter-alter interactions generate secondary ties]*

[School contact 1]

Of the choices below, who does *${q://QID19/ChoiceTextEntryValue/1}* interact with for information on teaching content and/or pedagogy?

[School contact 1]
- ☐ ${q://QID19/ChoiceTextEntryValue/1}
- ☐ ${q://QID19/ChoiceTextEntryValue/2}
- ☐ ${q://QID19/ChoiceTextEntryValue/3}
- ☐ ${q://QID19/ChoiceTextEntryValue/4}
- ☐ ${q://QID19/ChoiceTextEntryValue/5}
- ☐ ${q://QID22/ChoiceTextEntryValue/1}
- ☐ ${q://QID22/ChoiceTextEntryValue/2}
- ☐ ${q://QID22/ChoiceTextEntryValue/3}
- ☐ ${q://QID22/ChoiceTextEntryValue/4}
- ☐ ${q://QID22/ChoiceTextEntryValue/5}

[District contact 1]
- ☐ ${q://QID23/ChoiceTextEntryValue/1}
- ☐ ${q://QID23/ChoiceTextEntryValue/2}
- ☐ ${q://QID23/ChoiceTextEntryValue/3}
- ☐ ${q://QID23/ChoiceTextEntryValue/4}
- ☐ ${q://QID23/ChoiceTextEntryValue/5}
- ☐ ${q://QID25/ChoiceTextEntryValue/1}
- ☐ ${q://QID25/ChoiceTextEntryValue/2}
- ☐ ${q://QID25/ChoiceTextEntryValue/3}
- ☐ ${q://QID25/ChoiceTextEntryValue/4}
- ☐ ${q://QID25/ChoiceTextEntryValue/5}

[State contact 1]
- ☐ ${q://QID26/ChoiceTextEntryValue/1}
- ☐ ${q://QID26/ChoiceTextEntryValue/2}
- ☐ ${q://QID26/ChoiceTextEntryValue/3}
- ☐ ${q://QID26/ChoiceTextEntryValue/4}
- ☐ ${q://QID26/ChoiceTextEntryValue/5}
- ☐ ${q://QID27/ChoiceTextEntryValue/1}
- ☐ ${q://QID27/ChoiceTextEntryValue/2}
- ☐ ${q://QID27/ChoiceTextEntryValue/3}
- ☐ ${q://QID27/ChoiceTextEntryValue/4}
- ☐ ${q://QID27/ChoiceTextEntryValue/5}

[National contact 1]
- ☐ ${q://QID28/ChoiceTextEntryValue/1}
- ☐ ${q://QID28/ChoiceTextEntryValue/2}
- ☐ ${q://QID28/ChoiceTextEntryValue/3}
- ☐ ${q://QID28/ChoiceTextEntryValue/4}
- ☐ ${q://QID28/ChoiceTextEntryValue/5}
- ☐ ${q://QID31/ChoiceTextEntryValue/1}
- ☐ ${q://QID31/ChoiceTextEntryValue/2}
- ☐ ${q://QID31/ChoiceTextEntryValue/3}

[Continues to international contacts]

[page 10 was blank so deleted]

[The survey then moves to the next name that was supplied and asks the same questions]

Select the contact(s) from the list below that apply to the following question. Do not select the contact for themselves. Click the next button to proceed to the next question.

[School contact 2]

Of the choices below, who does *${q://QID19/ChoiceTextEntryValue/2}* interact with for information on teaching content and/or pedagogy?

[School contact 1]

- ☐ ${q://QID19/ChoiceTextEntryValue/1}
- ☐ ${q://QID19/ChoiceTextEntryValue/2}
- ☐ ${q://QID19/ChoiceTextEntryValue/3}
- ☐ ${q://QID19/ChoiceTextEntryValue/4}
- ☐ ${q://QID19/ChoiceTextEntryValue/5}
- ☐ ${q://QID22/ChoiceTextEntryValue/1}
- ☐ ${q://QID22/ChoiceTextEntryValue/2}
- ☐ ${q://QID22/ChoiceTextEntryValue/3}
- ☐ ${q://QID22/ChoiceTextEntryValue/4}
- ☐ ${q://QID22/ChoiceTextEntryValue/5}

[District contact 1]

- ☐ ${q://QID23/ChoiceTextEntryValue/1}
- ☐ ${q://QID23/ChoiceTextEntryValue/2}
- ☐ ${q://QID23/ChoiceTextEntryValue/3}
- ☐ ${q://QID23/ChoiceTextEntryValue/4}
- ☐ ${q://QID23/ChoiceTextEntryValue/5}
- ☐ ${q://QID25/ChoiceTextEntryValue/1}
- ☐ ${q://QID25/ChoiceTextEntryValue/2}
- ☐ ${q://QID25/ChoiceTextEntryValue/3}
- ☐ ${q://QID25/ChoiceTextEntryValue/4}
- ☐ ${q://QID25/ChoiceTextEntryValue/5}

[State contact 1]

- ☐ ${q://QID26/ChoiceTextEntryValue/1}
- ☐ ${q://QID26/ChoiceTextEntryValue/2}
- ☐ ${q://QID26/ChoiceTextEntryValue/3}
- ☐ ${q://QID26/ChoiceTextEntryValue/4}
- ☐ ${q://QID26/ChoiceTextEntryValue/5}
- ☐ ${q://QID27/ChoiceTextEntryValue/1}
- ☐ ${q://QID27/ChoiceTextEntryValue/2}
- ☐ ${q://QID27/ChoiceTextEntryValue/3}
- ☐ ${q://QID27/ChoiceTextEntryValue/4}
- ☐ ${q://QID27/ChoiceTextEntryValue/5}

[National contact 1]

- ☐ ${q://QID28/ChoiceTextEntryValue/1}
- ☐ ${q://QID28/ChoiceTextEntryValue/2}
- ☐ ${q://QID28/ChoiceTextEntryValue/3}
- ☐ ${q://QID28/ChoiceTextEntryValue/4}
- ☐ ${q://QID28/ChoiceTextEntryValue/5}
- ☐ ${q://QID31/ChoiceTextEntryValue/1}
- ☐ ${q://QID31/ChoiceTextEntryValue/2}
- ☐ ${q://QID31/ChoiceTextEntryValue/3}

[Continues to international contacts]

[process was repeated for all names provided. excluded here to save space]

Final Response

Having seen the questions, and acknowledging that your responses will remain confidential, please select whether you would like your responses to be used for research purposes.

- ◯ I agree and give my consent for my responses to be used for research purposes.
- ◯ I agree and give my consent for my responses to be used for internal Noyce evaluation but NOT used for research purposes.
- ◯ I do NOT consent for my responses to be used for research purposes or evaluation (results will not be submitted).
