# Supplementary material for: Social network data from teacher leader development
Source: Data Brief. 2019 Jun 24;25:104182. doi: 10.1016/j.dib.2019.104182 (PMC6617162; doi:10.1016/j.dib.2019.104182)
Supplement: Supplementary file 2 [file mmc2.docx]

Social Network Data from Teacher Leader Development

Polizzi, SJ; Ofem, B; Coyle, W; Lundquist, K; Rushton, GT

Appendix B: Activity Instrument

Navigating Teacher Networks

Perceived vs Real Power

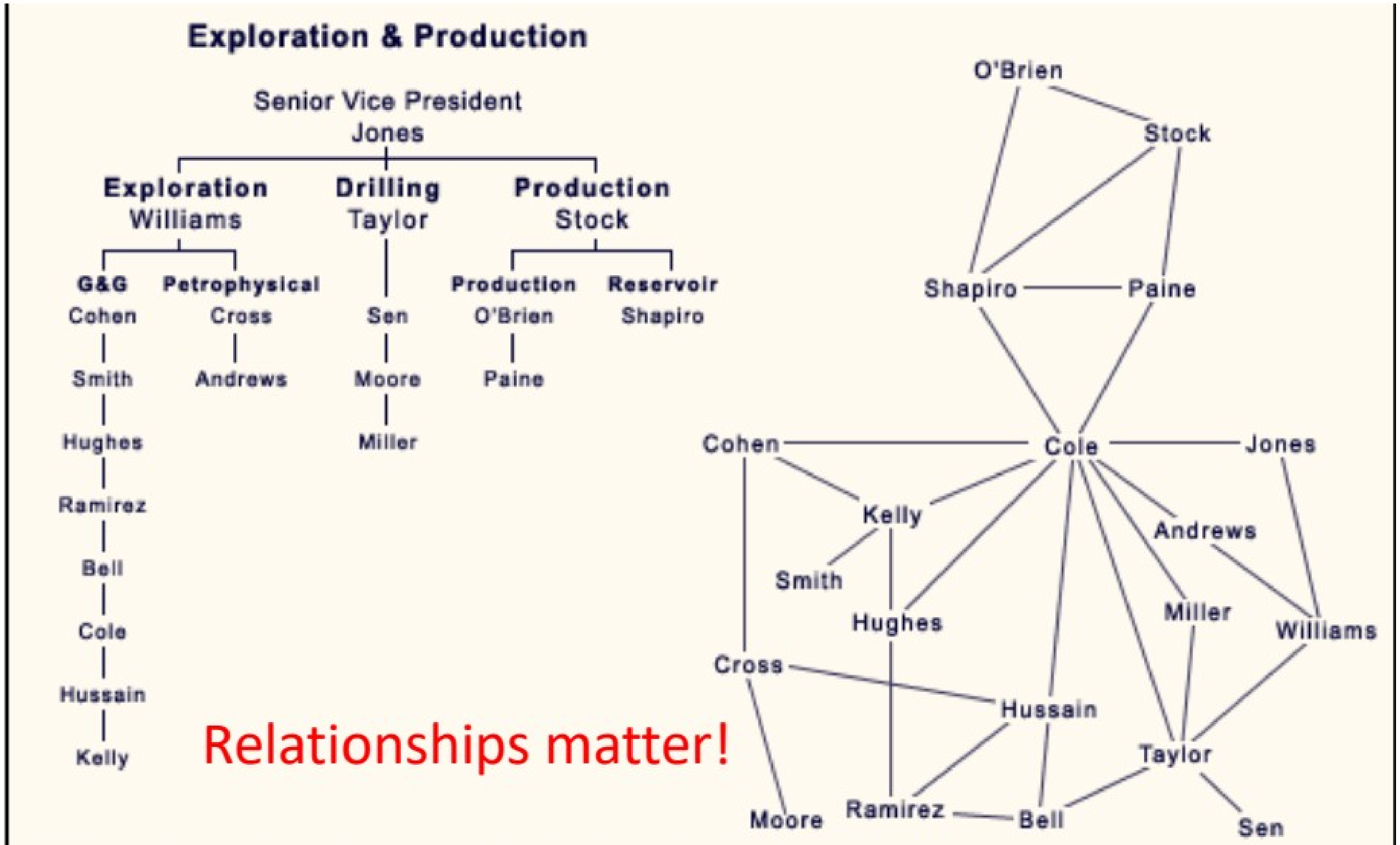

Relationships matter!

Source: Cross, R., Parker, A., Prusak, L. & Borgatti, S.P. 2001. Knowing What We Know: Supporting Knowledge Creation and Sharing in Social Networks. Organizational Dynamics 30(2): 100-120.

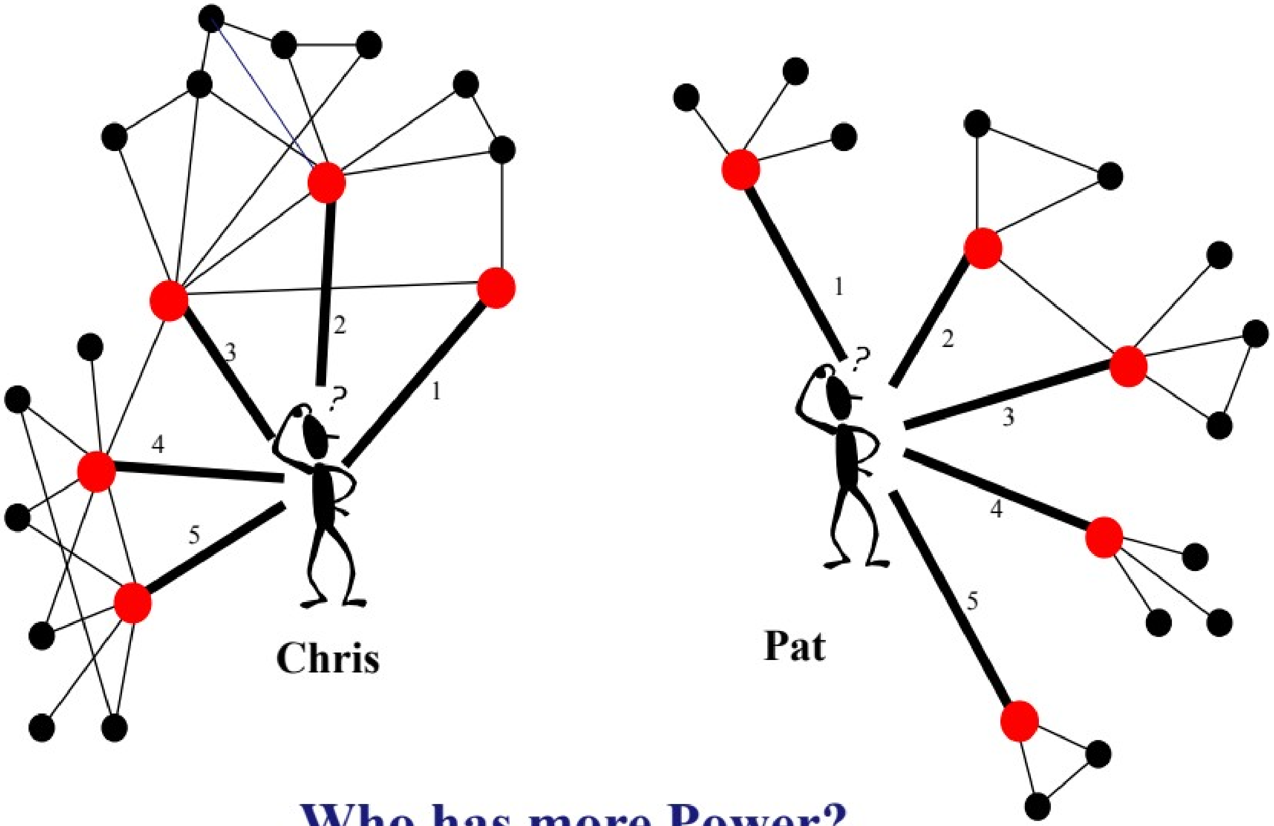

Who has more Power?

Constructing Teacher Networks

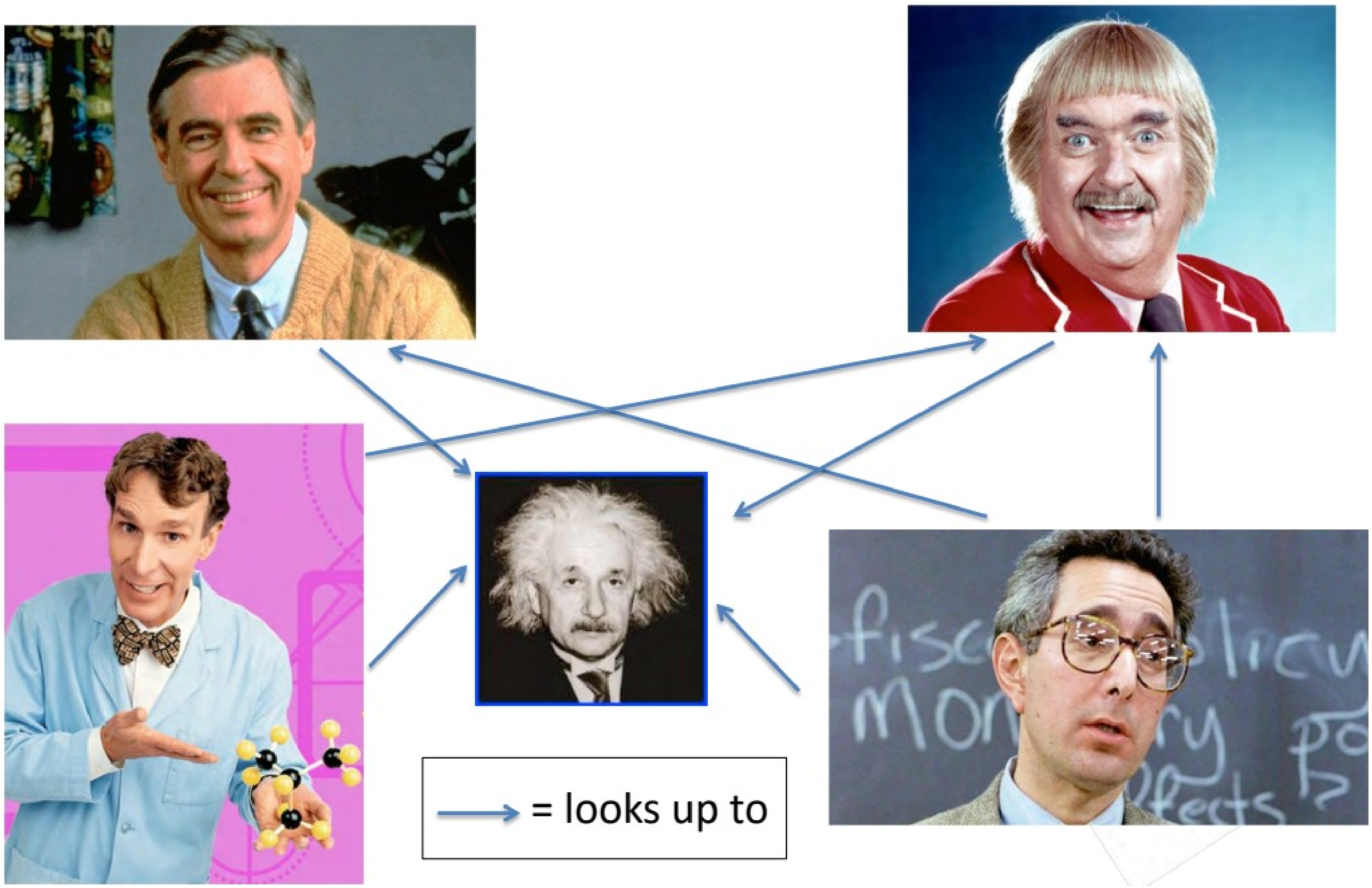

Survey

- Ego network
- Interactions for Information

- Q1: Demographics
- Q2: Who would you go to for information on teaching content and/or pedagogy?
  – school --district/county
  – state --nation
  – international

Identifying Social Capital

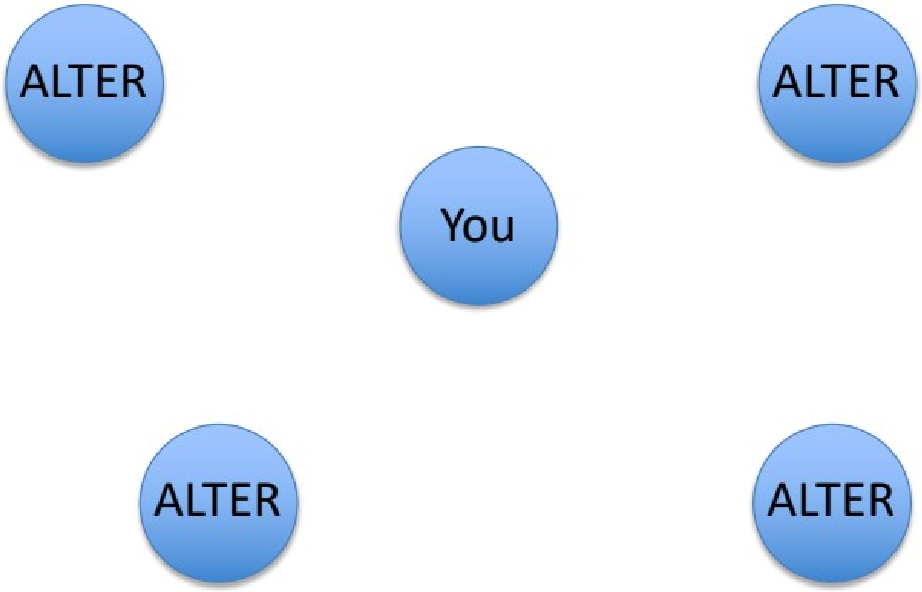

Survey

- Q3: Of the choices below, who does *School ContactA* interact with for information on teaching content and/or pedagogy?
  – School ContactB
  --District ContactA
  – State ContactA
  --Nation ContactA
  – International ContactA

Connecting Social Capital

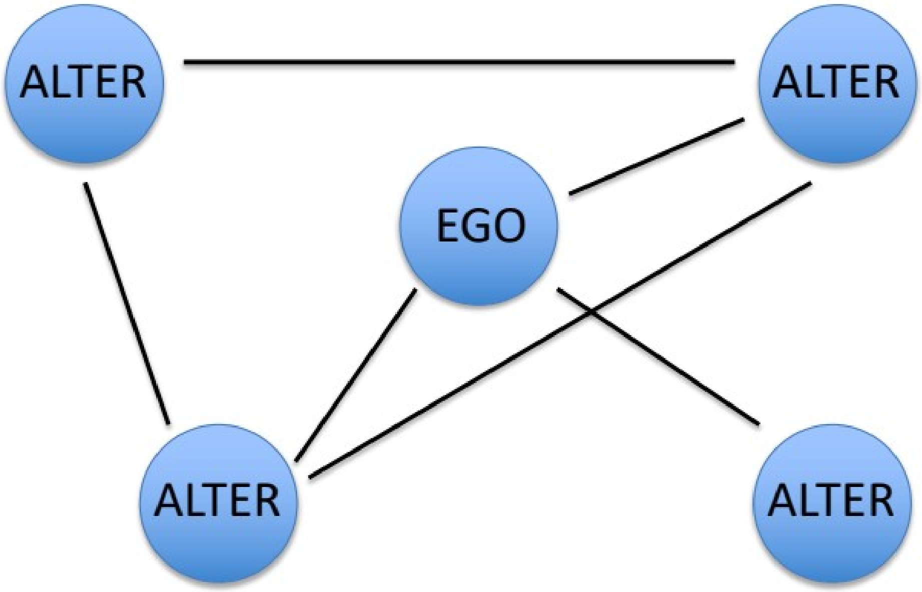

Big Reveal…

This is your map and not to be shared

This is your perception of connections

This is a snapshot from the day you were surveyed

Take a few minutes to become familiar with your network map

Closed Ego Network

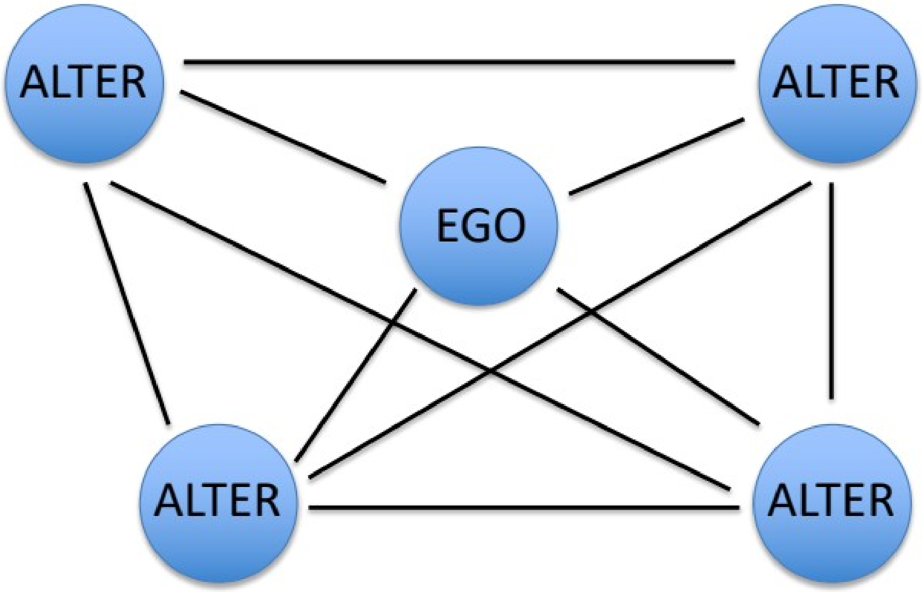

Closed Networks

Network Closure (Coleman 1988)

-Culture of obligations, expectations, norms
-Sharing of information, collaboration

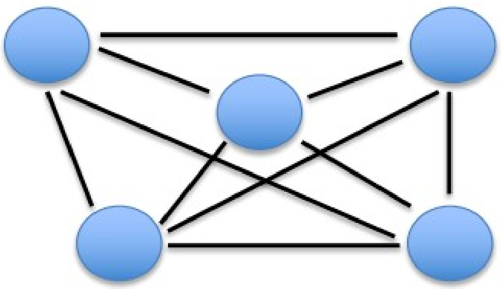

Density

-Conformity of ideas (Marsden 1987)
-Sense of inclusion (Brown 1990)
-Feelings of well-being (Bearman and Moody 2004)

Structural Redundancy

Open Networks

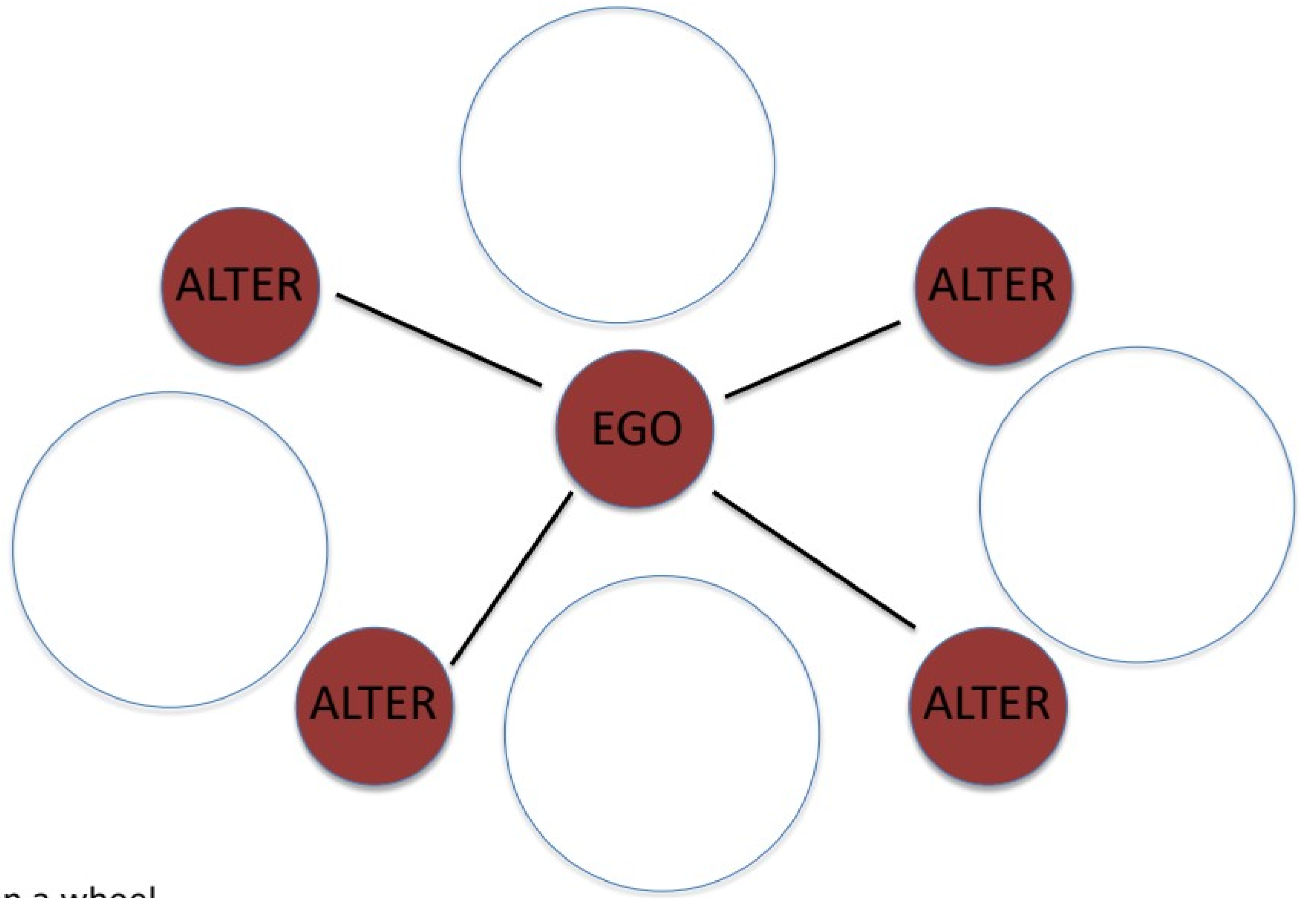

Spokes on a wheel

Ego Network Structures

Structural Holes (Burt 1992)

-Flow of information periphery to center (Burt 2000)
-Access to novel information/expertise
-New idea via synthesis (2004)

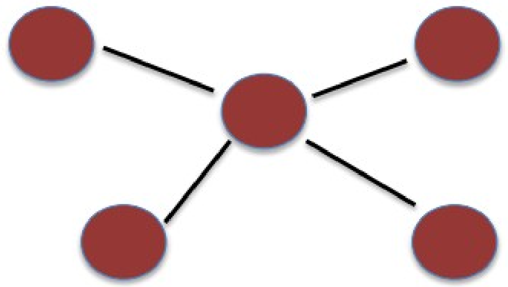

Ego as Broker

Scenario 1

Gamification of curriculum

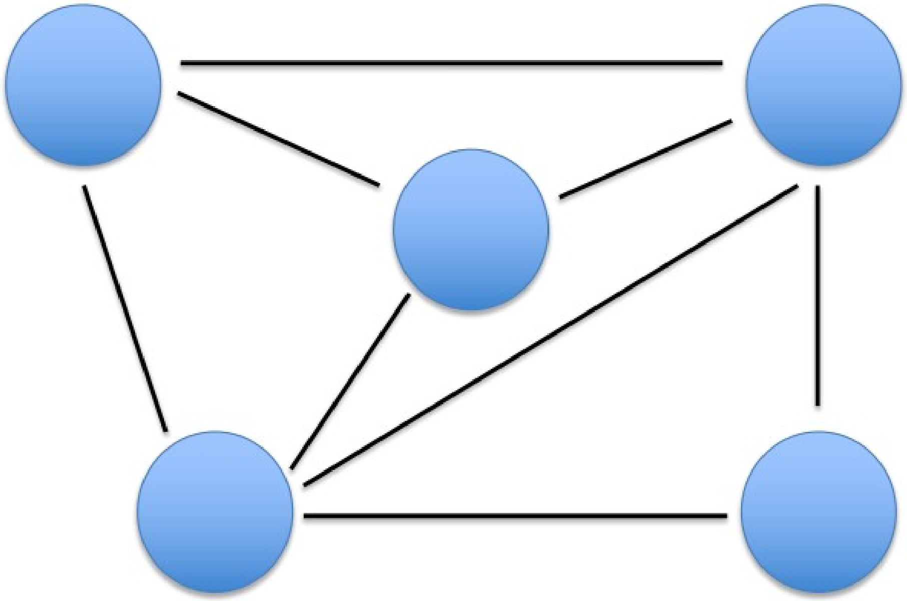

Scenario 1

Pause for discussion before moving to scenario 2

Scenario 2

Creating curriculum for out of field teachers

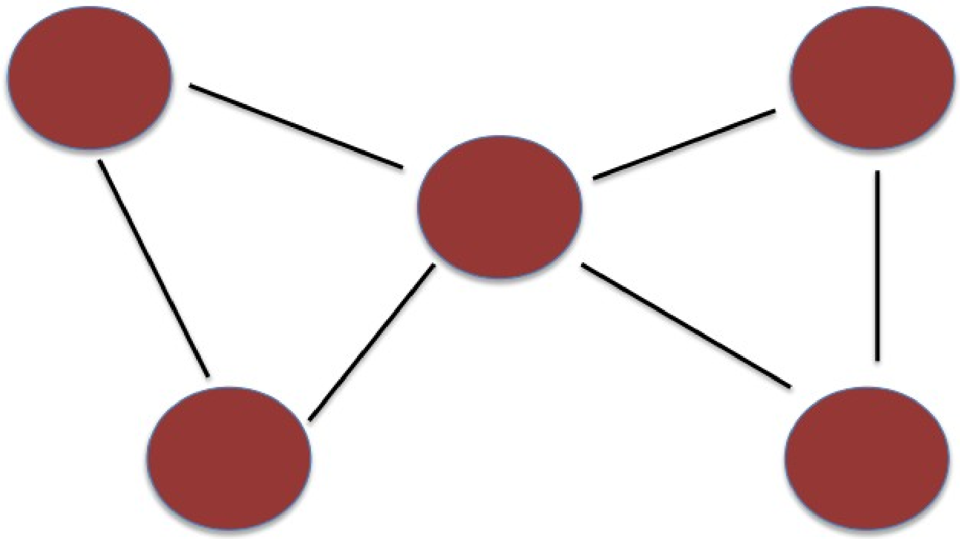

Scenario 2

Pause for discussion before moving to open floor

Open the floor to discuss any networks relevant to teachers

Pause for discussion before moving to hybrid

Teacher Leaders

- "…can increase other teachers' access to expertise and support, thereby creating a more collaborative work environment"

--Spillane and Kim 2012

(paraphrasing Lieberman et al. 1988; Weiss et al 1992)

HOW CAN TEACHER LEADERS HAVE NETWORKS THAT SHOW MUTUALLY EXCLUSIVE STRUCTURES?

Hybrid Network

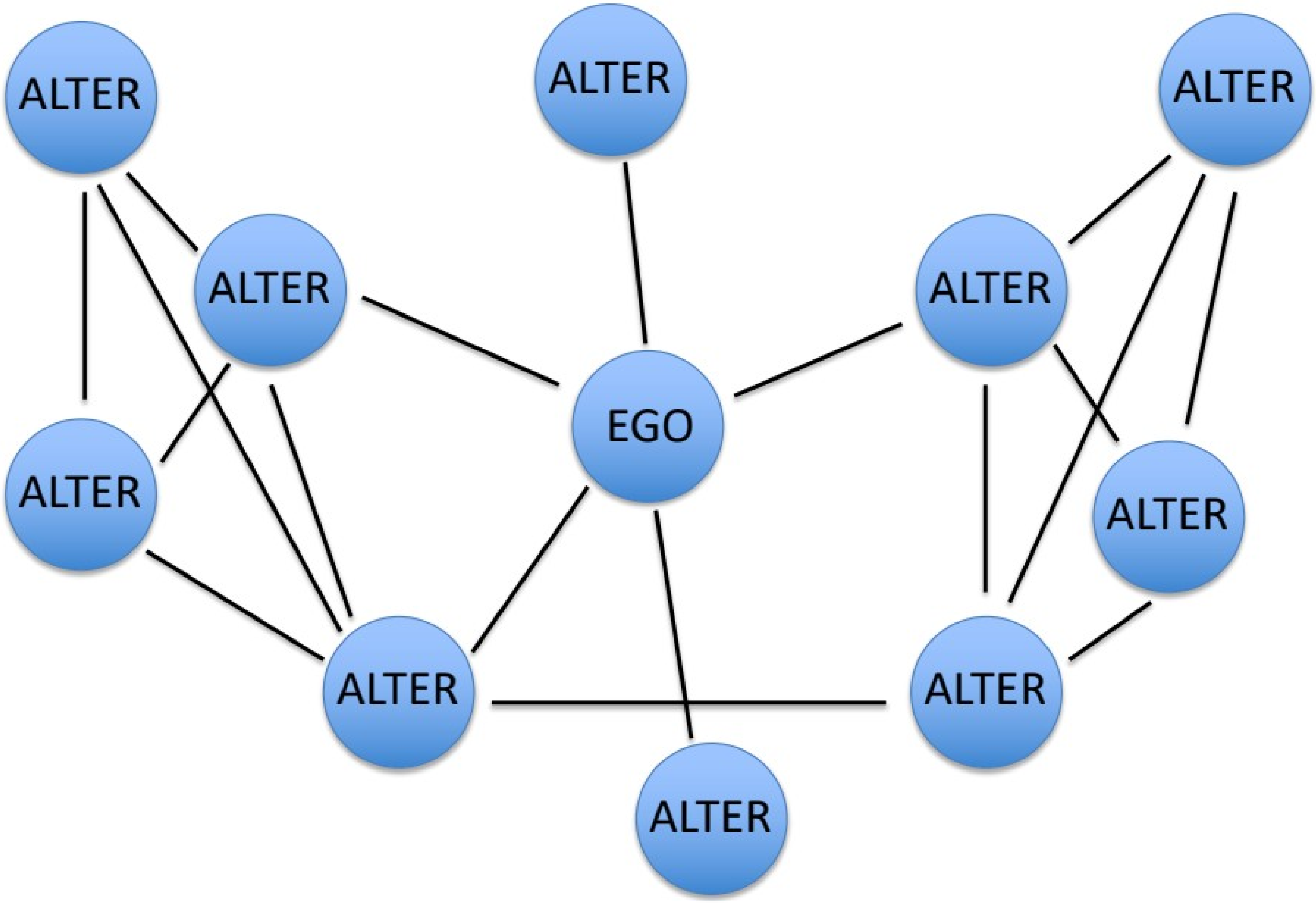

Involvement in Noyce

Can you identify your social capital?

Do you know where to get new info?

Do you know where to get support?

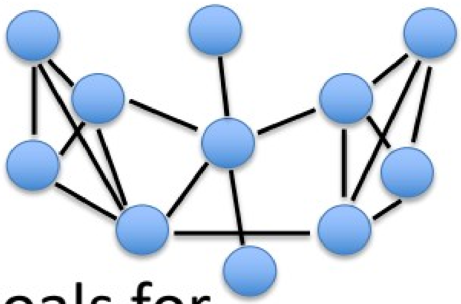

What do you need to achieve your goals for the Noyce program?

References

- Bearman, Peter S., & Moody, James. (2004). Suicide and Friendships Among American Adolescents. *American Journal of Public Health, 94*(1), 89-95. doi: 10.2105/AJPH.94.1.89
- Brown, B. Bradford. (1990). Peer groups and peer cultures. In S. S. Feldman & G. R. Elliott (Eds.), *At the threshold: The developing adolescent* (pp. 171-196). Cambridge, MA: Harvard University Press.
- Burt, Ronald S. (1992). *Structural holes: The social structure of competition*. Cambridge, MA: Harvard University Press.
- Burt, Ronald S. (2000). The network structure of social capital. *Research in Organizational Behavior, 22*(0), 345-423. doi: http://dx.doi.org/10.1016/S0191-3085(00)22009-1
- Burt, Ronald S. (2004). Structural Holes and Good Ideas. *American Journal of Sociology, 110*(2), 349-399. doi: 10.1086/421787
- Coleman, James S. (1988). Social Capital in the Creation of Human Capital. *American Journal of Sociology, 94*, S95-S120. doi: 10.2307/2780243
- Darling-Hammond, Linda, & Sato, Mistilina. (2006). Keeping Good Science Teachers: What Science Leaders Can Do (pp. 177-196): National Science Teachers Association.
- Marsden, Peter V. (1987). Core Discussion Networks of Americans. *American Sociological Review, 52*(1), 122-131. doi: 10.2307/2095397
- Spillane, James P. and Kim, Chong Min. 2012. "An Exploratory Analysis of Formal School
- Leaders' Positioning in Instructional Advice and Information Networks in Elementary Schools." *American Journal of Education* 119 (1): 73-102.
