# Supplementary material for: Social network data from teacher leader development
Source: Data Brief. 2019 Jun 24;25:104182. doi: 10.1016/j.dib.2019.104182 (PMC6617162; doi:10.1016/j.dib.2019.104182)
Supplement: Supplementary file 3 [file mmc3.docx]

Social Network Data from Teacher Leader Development

Polizzi, SJ; Ofem, B; Coyle, W; Lundquist, K; Rushton, GT

Appendix C: Transcripts Data

Facilitator: All right. So, as advertised, we have our first network–teacher network conversation, taking a survey, got a majority of responses back and had a chance to actually turn those into network maps that we can share with each other. I do want to tell you, before we get started, that I'm going to hand you your network, which may or may not have the name of the individual sitting right next to you, so my suggestion would be that you have at least one chair between each other, because these are networks that, you know, are characterizing your relationships, right? So, if for instance, I'm sitting next to [Person 1] and [Person 2], and I put down on my network that I interact a lot with [Person 1] and [Person 2 is] like, "Well, what about me?"

MS 2: Well, what about me?

Facilitator: Then, you know–

MS 2: [Unintelligible at 00:15:41].

Facilitator: Yeah. So–

MS 3: So, now feelings are hurt with that, right?

Facilitator: Now, you're getting [unintelligible at 00:15:47] in your network. Anyway, so, my suggestion would be not to share your [unintelligible at 00:15:53]. I'm only giving it to you–I'm only giving yours to you, so if you want to spread out a little bit that will make your part a little easier.

FS 4: What if the person is next to you and you don't have–

MS 5: I put Shelly on my [unintelligible at 00:16:05].

FS 6: An experiment just to see how close people are sitting and people who are close to the other network.

Facilitator: Great. Well, we will be [unintelligible at 00:16:15]. Okay. So, this is one of the slides that we showed you last time, right? It's on the left-hand side. They got an organizational chart. All right. And that may be the perspective of vice president or president, right? This is the way information flows in my network. This is the way organization is set up. That's not always the case. If you were to look at how organization works, right, like, who's sending e-mail to each other, who's exchanging correspondence within the company, you'll see that Cole is very essential in this network, right? So, relationships matter a lot of times in organizations more than the structure of who is a boss and who is reporting to who. Okay? So, taking that to the next step, we said–we started to look at these networks, the networks that actually exist in the

organization. Who has the most [unintelligible at 00:17:07], right? Is it simply going to be who is connected to the most number of people, right? So, in this case Chris and Pat [not participant names] are both connected to five people directly. So, you have to start thinking about the secondary connections, right, and connected to a very densely connected network or is it somebody that has fewer connections between better primary contacts, right? And so, we had some discussions about that. It may not always be one answer for every situation, right? Maybe context specific, what is that you're trying to accomplish and will work the best in what setting, right? So, we will go into some teacher networks, right? So, we're now talking about a shell–

FS 7: So, Captain Kangaroo–

MS 1: It is similar to kangaroo. So, we, we hear about teacher networks, right? So, we're not talking about the trust network or who's e-mailing who, right? It's what appeared on one of the previous slides. We're going to be able to define what everybody in here is thinking, right? So, it may be that Bill Nye the Science Guy looks up to Einstein, right? So, we will have arrows corresponding. Sometimes interaction and that interaction should be forwarded. Teacher specific [unintelligible at 00:18:20] network, right? And so, obviously, thankful for teachers that maybe gave in a network, even though, they did not exist or be on the same time period. So, what did we do? We gave you a survey. It's an ego survey in that it's about you, right? Your perspective on what your network looks like relative to someone else's network and what they think it looks like, right? So, you will be in the center of that network. Those arrows, what do the arrows mean? The arrows that we chose as interactions were going to be who is going to someone for information, right? So, this means that you're interested in getting information from somebody else. Okay. So, trust or we talked about love relationships or who's bowling with who, when we did the spread of innovation network. So, we started off by asking you some demographic information, and so, we can decide if maybe all of the ladies that we have in here are better networkers, right? They have denser networks than us, compared to the guys. And we can look at some differences like that that we won't really get into today. Question two is really how we start to build your network. You told us how many individuals you were connected with, and then, you were going to tell us kind of where they were geographically compared to you. Are you only interacting with the people at your school or are you interacting with the people on an international level? So, Greg went to Australia, so maybe somebody considers him to be international.

MS 8: All the time.

Facilitator: All right. So, in that first step, in that first step, we identified our social applicable, right? So, we talked about who's more powerful last time, and the power is going to come from all the people that you describe yourself with and how you use them, right, expanding our sphere of influence, getting more out of the people around us, ideas

of such applicable. So, you define all these alters, right, all these contacts that are just arranged around you in some fashion and they're related to you, right, so we expect to have some arrow connecting to you. But, we did something else beyond. We came back with a third question and said when you considered contact one, right? Does contact one interact with any of the other contacts that you were able to follow on the survey, right? So, now, we're going to start to build connections between the contacts that you've identified. All right? So, you would get up here at the center, because you are the ego, and then, we can sort of to say, "Well you identified Greg and Brett, well, they, in your perspective, may or may not collaborate together for that same type of information." Okay. So, that's why we start to build this up before we get you your own personal version. So, what I'm going to do now is I'm going to walk around and give you your networks. Again, this is your map. I would suggest not sharing it. It's for you. This is your perception of the connections, right? So, if we were to go through and, say, cull all of your e-mails to find out who actually went to someone else for information regarding the teaching [unintelligible at 00:21:33], we may get a very different look relative to how you perceived your network on that day, right? So, it's tied to your perception.

FS 9: Is that informative? Do you do that?

Facilitator: Do what?

FS 9: Do you look at what people–how people say they network and then how they actually network and . . .

Facilitator: So, you can compare, too? Yeah, you could. So, [Person 1], for instance, you experienced [Person 1] and [Person 2] working for [an external evaluator]. They work on some other large projects, and it's instead of doing the focus group interviews, they could go into company e-mail records, and they could just construct a network of who's interacting with who through that, so it'll be no survey to get–

FS 9: So, do you find that there are differences and what–are those meaningful?

Facilitator: So, from the research perspective, there are reports out there that suggest that if you have the actual network, right, from e-mails or something, right, and you compared someone's perception of that, the people who's perception is more aligned to reality get more done than the people who think there's something different, right? So, the executive may not be able to navigate that network very well, because they think it looks like an organizational chart, whereas, the secretary may realize that the network really looks like this, even though they are two very different [unintelligible at 00:22:58].

FS 9: Okay. Thanks.

Facilitator: Yeah. So, it's definitely something interesting to look at. It's not something that we're doing here right now. Okay. So, this will take a minute. I will pass it out, and you can start becoming familiar with your networks. And instead–just so you know, we've assigned pseudonyms based on just a random generator, so some of you gentlemen it looks like, kind of, female names. You enjoy that?

MS 10: And that's okay.

FS 11: So, if the people we mentioned also mentioned people, then you connected them?

MS 12: No.

Facilitator: No. So, what you're holding in your hand is based solely on the survey that you filled out, so if you identified working with [Person 1], me, and [Person 2], the follow-up question was does [Person 1] interact with [Person 2] or [me].

FS 11: Oh, okay.

Facilitator: If you said yes to either of those, there would be a connection between [Person 1] and whoever you said yes to.

Facilitator: All right. So, I do have–

FS 13: Ever?

Facilitator: . . . some additional information. It looks like we've had some networks that appeared kind of at the last minute, but I do not have the maps to hand back, right? So, if you did fill out the survey at home and I don't have a map for you. If you filled it out, probably, after Wednesday, then I'll have it for you.

FS 14: I filled it out last night.

Facilitator: So, now, you have your networks. So, like [Person 1] said what was his name? So, you're here in the center of that map and you were identified individuals who you would go to for information or content, right? I didn't say any other teachers. I left it open to any–whether it's professional, organizations. It could be people in the industry that you've connected with. It could be faculty and school. It could be anything, right? We didn't, we didn't constrain it that way. So, you picked individuals that you feel like you would go to. So, to me, what that means is that if you would go to them, you see them being, kind of, either at your level or above your level in expertise, right? So, I mean, they are some type of professional to you. Did anybody add to that individuals who might have been kind of, like, at a lower level than, say, on level with what you

consider to be your professional standing.

FS 15: Hm-hmm. [affirmative]

Facilitator: Did you put students on there or did you [unintelligible at 00:27:32]?

MS 16:No.

Facilitator: No? Okay. So, this is just me kind of taking the temperature of the room to see the types of individuals that you put on there. So, you personally know whether those individuals are at your school or at this basic level or anything like that. So, you can start to identify whether you have clusters that only exist at those certain levels, like, you have a cluster of people that are just your school and they're not connected to anybody else. You just start to see if you have clusters that are going to exist across those kinds of geographic grounds, right? So, maybe you are connecting teachers at your school to teachers here at [university], right, and trying to get them to work together. So, you're starting to consider that you have a bigger sphere of influence than someone who's not operating at, say, one level. All right? So, giving whether your network shape looks like versus patterns that you'd start to see in there and some people have going ahead and connected some additional knocks. Okay? I want to give you some classical examples of networks, talk about some things [inaudible at 00:28:39] with those networks, right? So, a closed network structure, right, we mean closed in that it's 100 percent density. What does that mean? It means that every single node, right, every individual in that network is as connected as they can be, right? So, you, here in the center, have four connections to the other four people in the network. Everywhere your contacts has more connections, right? There are no–there's no absence of connections. Every possible connection has been made. So, that is what we mean by a closed or dense network, and there are certain things that are associated with closed networks. So, network closure is one way to leverage your social cabinet. Maybe you were striving to have that super dense network. So, in a dense network, we end up with a culture of obligations. Can anybody give me one reason why we might have a culture of obligations and expectations in that dense network?

FS 17: You'd be surprised you guys didn't know about it.

Facilitator: Right. Everyone is connected and kind of visible at the same level. All right?

FS 18: With your family?

Facilitator: Yes. So, this could be a family scenario, right? There's actually–because this has been used in the social sciences, there was a study that found dense networks of families were more likely to reinforce the female role of cooking, and cleaning, and stuff like that,

right? Because there are expectations with your aunt or your mother and everyone else does it, and we're densely connected, and I say, "You're going to do this," and then, you have to say, "When," all right? There's that visibility of what your practices are and what your information is in a very dense network.

MS 19: That seems to pertain to teaching, right?

Facilitator: Yes, exactly.

MS 20: We, we have the same people who may not think about teaching ever differently, always going to think about it the same way, and you enforce that whatever we're doing must be good because we're all doing it that way.

Facilitator: Yeah. That's–yeah.

MS 21: Yeah. I just wanted to say something. I read [unintelligible at 00:30:55] at the university store about not hiring faculty form their own programs because they're trying to actually infuse their programs with extra ideas. And, you know, I used to think that's stupid, you should just hire the smartest people in the building [unintelligible 00:31:09].

FS 22: We do that a lot at our school, too. People are always surprised that we have so many brand new teachers, but we like the new fresh ideas that they bring, and if they have a lot of recent experiences in education, and so on.

Facilitator: So, the sharing of information collaboration is something that is typical of a closed network. Can anybody wager an idea as to why that would be?

FS 23: Can you ask the question again, sir?

Facilitator: Yeah. So, sharing of information collaboration is something that is associated with closed network. Why would that be the case?

MS 24: Well, it looks like we're all involved with each other versus–which is a much harder way to collaborate or share information than somebody up top saying, "Pass this on," for failing to change.

FS 25: I, also, think–just thinking about what it is. What is that black line represent? There has to be something that establishes that connection, that sharing process.

Facilitator: Yes. So, whether it's an e-mail, whether it's talking, in our case, we're talking about who would you go to, right? You'd feel like everyone is equally accessible. There is no problem, right? There is not a gatekeeper of information. There's not somebody up

on top, like what he just said. Yeah. If I don't get an e-mail from [Person 1], I have to, then, bypass him and go to someone else to get the exact or similar information. So, that structure then is going to give a function of collaboration. So, those are kind of some good things, I would say, in my perspective, at least. What else can density do for us? The dense network can also lead to conformity of ideas, right? So, if [Person 1] brought up the idea that may be the same idea just keeps getting passed around and passed around, and so, my network brought up that in order to get it out of that cycle bring in fresh blood, right? That's the phrase that you may not have associated with the structure of this, but having a fresh perspective, having somebody from the outside coming. So, a sense of inclusion, sense of inclusion in a network like that where everybody is connected on somewhat equal terms, so you might feel like you are truly part of the team when you have access to all these other people that have access to you. The only 12 names associated with that inclusion, usually if you feel like you're a part of a team, you're a valuable member, you are giving and taking and being respected, and you're going to have feelings of well-being, right? And so, when we talk about the sharing of information, it might be nice to be in a network like that, right, feel valuable, right, be the expert that's in that group that's connecting everybody, but sometimes, in order to get something accomplished, you just step outside of that comfortable little network that we've created and you try to be the one that gets access to these things. So, also, considering this, kind of good and bad, structure redundancy, everyone that knows this network is redundant, right? Each one of them has four connections, each one of them has connections to everybody else in this network. You take away this node, everybody still has the [unintelligible 00:34:38]. This group would probably still continue to function without that individual. Okay? So, when [Person 1] got up in the beginning of the meeting and said, "You know at some point the grant's going to go away, right," what kind of network do you have when you remove one or two individuals or some people here and that kind of stuff and you've created a structure that's going to be able to persist and you have a sense of one or two individuals. And so, looking at your own networks, do you see that it's completely dependent upon one person besides yourself? And so, do you want to shore that up and find other connections or just consider it?

FS 26: Does structural redundancy relate to working efficiency in any way? So, our very need to network some more of us efficient?

Facilitator: So, it would depend upon the text. You're trying to focus on the new, then, it may not be the best. If you're trying to get something done, then, sometimes you need enough hands to get something done.

MS 27: Maintain status quo, change status quo [unintelligible 00:35:50].

Facilitator: All right. Open networks. You may find that none of your connections were connected to each other or you may find some instances where you had single

connections kind of radiating like spokes on a wheel that were mixed in with a little cluster, like a little dense group. Okay? So, this is something that we call structural holes. We'll say that your network has structural holes, and that's because that in this network structure, you have holes where you could have had connections, right? So, what are structural holes mean for a network? So, if you have structural holes in your network, that means that information is going to have to flow in certain paths, right? It's not every road possible, but there are certain roads through which information is going to pass. So, if somebody has an idea over here, it's going to have to go to the ego in the middle. It's going to have to go to you, and then, you're going to decide whether you're going to disseminate to the other people in your network if it's a good idea, or if it's related to what you're working on. Or maybe you have a contact over here and he's working on something, and [unintelligible 00:00:08] want to do something, and you say, "Well, maybe that won't work, but I know somebody who would be interested in this." So at that point, you are in between them, and you could help facilitate a connection that goes directly from one end to the other.

So, access to novel information. Well, we said with that super-dense network that one idea may float around an entire group, it's less likely that you'll have that structural redundancy here. Right? These don't normally, in your perspective, speak to each other in the same capacity about information [unintelligible 00:00:46]. So it's more likely that these people are sources of that novel expertise—novel information, different sources of expertise.

It might be that you've got somebody's who's really good at differentiation working in a certain school district, and you may have someone at the State level who's working on [DVL? 00:01:08]. Right? So you then are able to ask a question here, ask a question here, ask a question here, and then synthesize the data when it comes into you, and do something new.

So the idea of using the social path of what's available to you in these situations, where you have structural holes, is that you can synthesize new ideas. You get novel information, and then create something new. Okay? So take what you want from different perspectives; do whatever you want with it here in the center.

That means that you, in the center of whatever those spokes on the wheel are at, if you're in the center of that wheel, you're going to be able to act as a broker, so it's like a brokerage position.

Some of you may have personalities that make you think, Yeah, I want to be in the middle. I want to play these people off each other, or I feel like I'm empowered enough to connect people; be in between.

Some people don't like being in between, so if you were the only person holding a gigantic network, you may feel like you're being pulled in one direction and then being pulled in a different direction.

So this comes to your locus of control—or, your sphere of influence, excuse me. Where do you feel like your strengths lie? As we move from that comfortable zone, maybe it's time to start looking at how I can leverage different parts, instead of just being one part, one cog in the wheel.

MS 2: So the leadership thinks two aspects of leadership that are really kind of critical or the focus of our project next, innovation and empowerment. So I see kind of strengths in both kinds of networks, right? The open one is likely to generate innovations in your network. You may be an innovator if you're connected to a lot of these great social resources. And if you want to empower people, that is likely that schools get them to work together and get them to then, you know, interact with others then having some closed network aspect of your work is probably something I would be looking for.

But it's what I'm talking about is do you think that's consistent with understanding social networks, or not?

Facilitator: So I want to reiterate that we've just got a snapshot. Right? So when you took that survey, that's the way that you received it. Since then, you may have gotten together with a PLC, or you may have a project with someone—[Person 1] said that she wanted to come up and talk about that at some point today.

So they have started to engage in another project. Right? So they may have taken two people that we didn't originally see as connected and say, "Let's get this together and start a taskforce." Or somebody you heard is motivated to do some change.

So I think that this dynamic is going to be tailored to whatever situation it is that you're working on. Right? Now that you have these maps, you can say, "I don't normally see these people as being connected, so maybe I want to bounce ideas off these different people who aren't connected when I want to get something done. Maybe I want to move to this group that I know works well together, and shares ideas, or are on similar playing fields." Does that answer your question?

All right. So I just brought up PLCs. I want to pose this question to the group then. If this is the network that you have, and you want to implement gamification of your curriculum—right?—starting from ground zero, do we think this is a good or bad network; or, what features of this network are going to make this successful or not, if you know nothing about gamification and you just decide, I want to start incorporating this in my classroom?

FS 3: If everybody else is into doing the same thing, it might be effective to work together, you know, each of you can research different parts and bring it together into a program.

Facilitator: Okay. So—

FS 4: It's not open, though. Like that spoke, like you're going to add a dot to that.

FS 3: Assuming that nobody in your network already [unintelligible 00:05:47].

FS 4: Right.

MS 2: Well, if you're trying to implement gamification, then it's likely that it doesn't currently exist or else he wouldn't have drawn it that way. It's likely that that doesn't have gamification in it presently, or else he would have indicated that.

FS 3: Or you've never talked with those people about it.

MS 2: Well, then—

FS 4: Who are your people?

FS 3: I mean, I don't think I can think of a scenario where someone violated a very closed network at work.

MS 2: [Unintelligible 00:06:19].

FS 3: I do know that.

MS 2: Right. Right. So…I think I agree with you, Becca, that it would—Rebecca—that it would probably [have access issues? 00:06:28].

Facilitator: Okay.

FS 3: Then it seems like you might have to get agreement, get everybody to agree that it's a good idea before you can move forward. Maybe—I'm just thinking about this from a standpoint of working together in teams toward a common goal and unless everyone is sharing, or if everyone doesn't think this is a good idea, but everybody needs to be doing the same thing…

MS 2: Like it may be difficult for them just because of cultural norms that are [unintelligible 00:06:59].

FS 3: Right. There's already an established way of doing things [unintelligible 00:07:05].

FS 4: Everyone has to buy in.

FS 3: This is how I feel about information that I [unintelligible 00:07:17]. And then I'm—

MS 5: You're [unintelligible 00:07:18].

FS 3: Right. Well, I'm—well, yeah. I'm the dot in the corner. I'm the purple dot, but I've got this connection to the green dot that nobody else has. The green dot is all that's useful in

all the information that we collectively have, then I am connected to Noyce and I'm the only one connected to green, and anything I bring back I can share with the school.

MS 5: You have to figure out a way to share it to hopefully get everybody excited about it. Because with a group that's interconnected like that, they're already talking to each other about it, so if you don't communicate it to that group in a way that gets them all excited about it, you know, it's not going to happen.

Facilitator: Or through the innovation simulations that you went through, maybe you know that this person is really happy about everything; and you can get them on board, and systematically kind of motivate the group. Right?

FS 3: Have lunch with somebody, right?

Facilitator: So, yeah. So if you don't know your network, knowing what it looks like or how people are connected, you might be like, "Yeah, I can start a few steps at a time." If that's what you want to do.

Because I brought up social norms, like it might be a good thing to get something accomplished, but Amanda is bringing up that there may be this structure in place, if this is all happening at your school—right?—at the school level, then you have to combat that in order to bring in the novel information.

Okay, so that was just one scenario. It was all with a closed network.

So we've got another PLC that I've heard talked about. It's that we are going to, we go to meetings every year, and there's always a biology teacher who is coming into the physics classroom for the first time. Let's try to create some curriculum that is going to target them, and then we're going to try and implement that overall.

So my question is, for this type of network—this is some portion of the network that you've had in front of you for the [unintelligible 00:09:24]—what are the good or bad aspects of this network for trying to establish that goal or get to that resolution?

MS 5: Now you have to individually convince every single one of those people that's the way to go. Because they're not going to reinforce each other at all if they're not talking to each other. So you now have to have four individual transactions to convince them of this.

MS 2: You know, that's a dissemination idea. I think creating the curriculum, this is probably a good way to do it—right?—get all these different ideas out. No?

FS 3: Doing something similar with the GPB stuff, like it's been amazing to be able to negotiate with other people about, "Oh, this is important to chemistry, for example, because of this, and we should do this in this order." So I just think having one person then trying to convince, okay, so the person down in the corner thinks that we should do it this way, so now I'm going to talk to you other three about it. But you have a different

opinion, so now I need to go back and talk up here, and then I've got to go back and talk to everybody again to try to negotiate a common set of—yeah, vocabulary, or the words that we use to say that these are the most important points of the physics curriculum that I would feel teachers need.

So I think that this would be difficult. It might be a good way to, once you have the agreement, to then divide duties. Like, okay, this person is going to go work on, I don't know, a physics thing, and it's all [unintelligible 00:10:48].

MS 5: This brings—

MS 6: Or if everybody on the outside believes that person in the middle is a goober, and they'll do whatever they tell them to, then I guess that could be effective. Hopefully that person in the middle knows what they're talking about.

MS 3: Well, if you think that person in the middle is a moron, you've got a big problem.

Facilitator: Any other perspectives on whether this would be good?

FS 4: Well, the dot in the middle needs to introduce some of the dots on the outside. They don't have to all be like—that needs to be a star.

FS 3: Or at least a bowtie.

FS 4: I didn't even realize—yes—I now realize that I didn't click some things, I think, when I did my survey, and there were some connections on mine. Mine doesn't look closed and it doesn't look open, it's like this star crisscrossed bowtie triangle business. I didn't factor in these extra connections.

Anyway, but I think that with some introductions between some of these people that we could get things done; but also it adds to, it would add to conversation, that new perspective. So I think that the dot in the middle needs to have a party and invite all the other dots [unintelligible 00:12:12].

Facilitator: So if this for out of field teachers, and this person here is physics, maybe they know someone who's chem and maybe knows at an someone who's environmental, and, you know, biology or whatever. And so maybe these people normally don't talk to each other, but they're the one who is going to get feedback from all these different groups, and then synthesize what needs to happen in order for the out of field teachers to feel like they are being addressed. Right? Because they have a background in environmental or chemistry or something else, so they may need to have these novel sources of information as they can get, synthesize a new outcome.

But then, in order to—and that's the creation part—but then in order to implement it, you may need a group of other likeminded physics teachers who can leverage all this

information to come up with a consistent idea of what should happen at an AAPT meeting.

MS 7: I was going to say something along those lines. If I'm in charge of creating curriculum, then I want a network that looks like that, where I can pull from lots of resources that are going to have different information. I wouldn't want a closed network where, if one person doesn't know it, no one knows it. But if I was trying to get something done as a group, I'd probably prefer the closed group, where we're all pretty closely tied to each other and work well together.

Facilitator: So one perspective on teacher leaders that doesn't come from a person talking about social networks is that teacher leaders are going to "increase other teachers' access to expertise and support, thereby creating a more collaborative work environment."

So, since they never wrote that intending for someone to think about it from a social network perspective, we have two very distinct network structures that come out of that comment. I showed you the open network, in these kind of maroon-reddish spheres; and then we talked about that "collaborative" network that's going to appear in the blue or purpley kind of color network. Right? A dense network.

So how is it that we end up these mutually exclusive structures if the examples that I gave you were just an open network and a closed network? One possibility is the idea that we have kind of like a hybrid or a mix between the two. And so we may have a group of contacts that are over here on this side that are well connected to each other. This may be a group that is working to try and create something or get a job done. Same thing over her. This might be somebody else, some other project that you're working on [unintelligible 00:15:05].

But at the same time, you maintain some contacts that aren't really related to anybody else. These are your fresh perspectives. This is experts who may know something about gamification, and can give you ideas, and you can leverage their resources and things like that, but you don't necessarily need them to work on the day-to-day activity of trying to get that curriculum designed.

You may design over here and then shoot it over here and say, "Hey, does this make any sense?" So you've got a fresh set of eyes thinking on things.

You, here in the center, are going to be able to decide how the information flows. Right? You can pull it in. You can send it to one of these groups, or you could send it to someone else. You could send out multiple feelers to come up with good ideas of what the out of field teachers need, because you've contacted a biologist and a chemist at the same time. Right? We don't have to go sequentially. This is not a network that is only tied this way, where you have to wait for So-and-So to talk to So-and-So. You're actually connected to those knowledge sources.

So with your involvement in Noyce, why are we doing this? Just to kind of bring it home. So can you identify the social capital that you have available to you? I didn't get a single survey that was blank, or had less than probably five notes. So you know of teachers around you at some level—at your school, or state or something—and they have some kind of information that you feel is valuable enough for you to think that you want to go to them. It doesn't have to be constant, it could have been [unintelligible 00:16:58]. So they're at your level, they're above your level, but you still feel like you can go to them. So that's great. You do have some network support around you, and you at least know about it so you can think about tapping into it.

So where are we going to go to get new information? We're probably going to look for people who are less connected to the rest of our network. Right? They would be kind of the spokes on the wheel. They would be those fresh perspectives.

If you don't have any fresh perspectives, and you are engaging in a new project or some new endeavor, it might be time to think about the last conference that you went to, or to peruse Web sites of schools around you, to try and think of who might be able to help.

FS 8: What I was thinking about with these questions was that this didn't tie into anything like the larger network. Like, you know, where would I go to get new info or new ideas would probably be to go to a conference, which would tie me into a whole other set of networks. If this is only individuals, not like connected to organizations, where you could get a whole lot of—

Facilitator: Hm-hmm [affirmatives].

FS 8: Like what we were talking about with the AP stuff earlier, the AP online resources came up. That's like that's a place to get some support as well as the new ideas. So that's something else to consider as part for your overall network, or the other network you can tap into.

MS 2: We've seen in other sociograms things other than people, and their [unintelligible 00:18:29] spheres [unintelligible 00:18:31] entities.

Facilitator: Yeah. So you're at least thinking about, do I have limited information in my network, or should I be looking somewhere else? Whether it's an organization or whether it's a person.

You know where to go to get the support that you need, the bodies to get something done. Is that going to be at your local level, or do you have a group that's well connected that's operating at the state level that just kind of work well together and is connected, and you're able to divide the task and come back together and get something done?

And then, what do you need to achieve your goals for the Noyce program? Do you have a dense network that is missing some external content, contacts? Are you at the phase of your project or goals where what you need to do is get a group together that's all

interested in the same thing, and wants to make curriculum, and wants to videotape it, wants to share it? And do you need to find those likeminded individuals.

So. [unintelligible 00:19:40]. Look at your network. There is a lot of information there that may help you think about the interactions that you have, whether people are resources for ideas, and maybe a larger geographical range, versus people who are physically hands-on helping you get something done at your school. Okay?

Any questions or comments or other projects that you feel like fit into social networks?

MS 2: This is somewhat of a kind of a self-assessment tool, and what I'm hopeful is that you'll see how you perceive your network functioning, and that can probably form why you're able to do some things or not, and then maybe you could see what you need in your network. And then we'll be able to watch, as you change, like what happens to your network? Like we think that leadership is going to be somewhat able to be measured through these social networks. I mean, what is a leader? It's someone you can see what their influence is on others—right?—over time. I mean, that's one measure of it.

So we're interested in seeing—say, at the end of this project—does your network look any different, and do you look any different in your sphere of influence now? [Unintelligible 00:21:09].

MS 9: Do I need to go through or something [unintelligible 00:21:18] and just redo it and like seriously like?

Facilitator: Well, no. I don't want, for instance, everything we talked about now to completely influence the way you create your map. Right?

MS 9: No. Every time [unintelligible 00:21:32].

Facilitator: So you're listed on my pseudonym list, so I think you submitted it. I just don't think we've had time to crunch it before this meeting.

Facilitator 2: I want to bring up a question. So Kentucky is implementing NGSS, and in order to do that, they developed a model where they were going to construct a teacher leader network. And so the idea was that each school in the State of Kentucky would get to nominate two teachers, two or three teachers for the network based on different grade bands. They would go to regional trainings, and then they would take that information back to their school and [unintelligible 00:22:13].

Now, the group that's doing this at the Department of Education feels like this model is a rousing success. The teachers that I'm talking to [laughter] do not see it as a rousing success. And so I wonder, with our discussion we had, why do you think, as a teacher yourself, where that model might go wrong? Or how might it be constructed to be more effective?

FS 3: Who chose the teachers that are going to that?

Facilitator 2: Door number one: the administrators, not the teachers. The criteria was [unintelligible 00:22:56]. Like what would be the characteristics of the person who is chosen to be in that teacher leader network?

MS 5: [Unintelligible 00:23:07]?

Facilitator 2: Yeah. Like if you're choosing in really good, logical criteria, instead of administrators probably doing it in haphazard ways…?

FS 3: Well, if someone's excited about it, and an innovative teacher, and they want to change, not because [unintelligible 00:23:22] tells you this is something that you have to do. Somebody who actually does some of the things, like who has dynamic lessons and stuff in reality, not on paper.

MS 7: And who already has the respect of the other teachers.

MS 5: Somebody you already want on it.

FS 8: Well, also. I think that sometimes you have really amazing teachers who may not be noticed so much. So it may be the administration needs to ask department chairs for nominations, and then, you know, ask that teacher a little more to do stuff. I mean, recently, I've been nominated to do stuff, and they are things that I didn't necessarily set out to do. I was like, "Okay, I'll do that," and then interviewed for it.

So I think some of it is when you just appoint teachers and like, "Oh, and I get a stipend!" And, "Oh," whatever, they might not be the best choice. So do more of a nomination/application type thing, so they have to ask them [trails off 00:24:38].

MS 5: What I wonder, too—what I have in mind is something that got brought up in this project a number of times, that some of you said it's easier for you to function as a leader outside of your school than it is in. A couple of you have said that. I don't know how many of you feel that, sense that; but if you could tell us why you said that? If you do sense it, why do you sense that, or how we can address that condition?

FS 3: I think it's a hierarchy that's at school, and if you're newer to it, you haven't worked your way up the hierarchy to be in that position. But on the outside, it's easier, you know, like if you've got a whole bunch of strangers coming to a place, it's much easier to interact or whatever because there's none of that in place.

MS 7: And you, the outsider, are dealing with people that are interested in that topic, whereas at school, it may be forced upon everybody.

FS 8: And then even if the changes are forced upon everybody, you may not agree with the other people around you, but I'm going to do it because I'm going to be the catalyst that goes in there [unintelligible 00:25:43].

FS 3: I think this conversation, what you made me think of, like if you're only sending two people from each school, you're not making—you're not interconnecting enough people to other people. And I realize there's a cost function to that…maybe it's not possible to send 10 people from every school, but then you take, you know, 10 people from every school with 10 people from all these other schools, and you have interconnected all those people with all of those people. So then when they come back to the school, there's not just one or two people as a source of information for the whole rest of the school. Then there's a lot of people who have each other—those 10 people or whatever—and now, if I'm not one of them, I have lots of people I can connect to who are then connected to lots of other people as far as sharing ideas and things like that.

MS 5: So if you don't have the budget to get some people from every school, you could pick a few schools. It's better to be effective in a few schools than not effective in any schools at all. And that could grow from there.

MS 7: And I feel like [unintelligible 00:26:47] Georgia has this certification thing going on, just how to spread that, and what kind of network Georgia might set up to spread that effectively.

Facilitator: All right. Thank you all for your attention.

[Applause.]
